# Melt Alloying of Two-Dimensional Hybrid Perovskites: Composition-Dependence of Thermal and Optical Properties

**DOI:** 10.1021/jacs.4c12697

**Published:** 2024-11-22

**Authors:** Arad Lang, Celia Chen, Chumei Ye, Lauren N. McHugh, Xian Wei Chua, Samuel D. Stranks, Siân E. Dutton, Thomas D. Bennett

**Affiliations:** † Department of Materials Science and Metallurgy, University of Cambridge, 27 Charles Babbage Road, Cambridge CB3 0FS, U.K.; ‡ Department of Chemistry, University of Liverpool, Crown Street, Liverpool L69 7ZD, U.K.; § Cavendish Laboratory, University of Cambridge, JJ Thomson Avenue, Cambridge CB3 0HE, U.K.; ∥ Department of Chemical Engineering and Biotechnology, University of Cambridge, Philippa Fawcett Drive, Cambridge CB3 0AS, U.K.

## Abstract

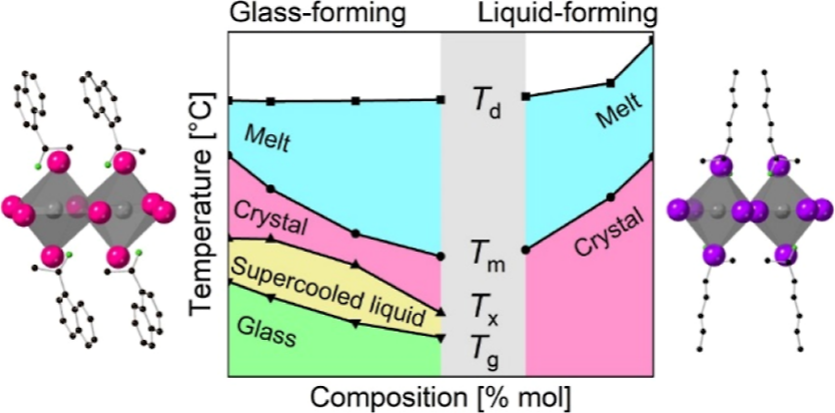

Melt alloying, the process of melting a physical powder blend to create a homogeneous alloy, is widely used in materials processing. By carefully selecting the materials and their proportions, the physical properties of the resulting alloy can be precisely controlled. In this study, we investigate the possibility of utilizing melt alloying principles for meltable two-dimensional hybrid organic–inorganic perovskites (2D-HOIPs). We blend and melt mixtures of two selected 2D-HOIPs: the glass-forming (*S*-NEA)_2_PbBr_4_ (*S*-NEA = (*S*)-(−)-1-(1-naphthyl)ethylammonium) and the liquid-forming (1-MHA)_2_PbI_4_ (1-MHA = 1-methylhexylammonium). Upon melting and cooling, 1-MHA-poor blends (*X*_1-MHA_ ≤ 50% mol, where *X*_1-MHA_ corresponds to the relative molar concentration of (1-MHA)_2_PbI_4_ in the blend) form a hybrid glass, while 1-MHA-rich blends (*X*_1-MHA_ ≥ 70% mol) crystallize. The melting temperature of all blends, as well as the glass transition temperature of the glass-forming blends, change according to blend composition. In all cases, melting produces a homogeneous structure, either glassy or crystalline, which remains such after the glassy samples are recrystallized upon a second heat treatment. This method enables band gap tuning of the blends, given that it varies with composition and crystallinity. Overall, this work demonstrates the applicability of classical melt processing to binary-component functional hybrid systems, and paves the way to solvent-free perovskite-based device fabrication.

## Introduction

Melt alloying, also known as liquid-phase mixing, is a fundamental concept in the field of classical metallurgy. During the alloying process, two (or more) metals are mixed and heated above their melting temperatures, followed by subsequent cooling of the melt.^1−3^ The resulting alloy possesses physical properties which differ from the ones of the original metals. This process has been shown to affect the electrical conductivity,^4,5^ the thermal conductivity,^5,6^ and the mechanical strength^7−10^ of metallic alloys. In all cases, the altered properties are primarily determined by two factors: (i) the identity of the constituting metals, and (ii) their relative concentration in the blend.

The concept of melt alloying has also been successfully utilized in the field of polymer science. Different types of polymers were shown to blend upon melting,^11−13^ and some even demonstrated phase separation when cooled down.^14^ Polymer melt blending has been used to enhance the materials mechanical^15−17^ and optical^18−20^ properties. Polymers have also been combined with inorganic components, such as inorganic nanoparticles (NPs)^21−23^ or carbon nanotubes (CNTs),^24−26^ thus utilizing the functionality of the inorganic component while maintaining the mechanical flexibility of the polymer. Such combinations allowed the formation of new hybrid (organic–inorganic) structures. Recently, liquid-phase blending was also applied to glass-forming metal–organic frameworks (MOFs)^27^ and coordination polymers (CPs).^28^ This, together with the previously demonstrated mechanical blending,^29^ paves the way for new postsynthetic alloying of hybrid materials, in which each component is itself hybrid.

Two-dimensional hybrid organic–inorganic perovskites (2D-HOIPs) have recently been explored for their use in optoelectronic applications.^30−32^ 2D-HOIPs are built from layers of corner-sharing [MX_6_]^4–^ octahedra, where M is a divalent metal cation, most commonly Pb^2+^, and X is a halide. The inorganic layers are separated by large organic amine cations.^31−34^ When the chemical structure of the 2D-HOIP, and especially of the organic cations, is carefully engineered, these materials can undergo melting at relatively low temperatures, before decomposition.^35−41^ Such melting opens the door for a solvent-free device fabrication route, which could potentially increase the resulting device metrics.^42^ It was recently shown that some specific meltable 2D-HOIPs tend to form a glass upon cooling, rather than recrystallize back to their thermodynamically stable crystalline phases.^40,43^ These so-called melt-quenched hybrid perovskite glasses are a new class of materials, which joins coordination polymers (CPs) and MOF glasses^44−46^ under the broader category of “hybrid glasses”, separate from existing organic, inorganic and metallic glasses families.

In this work, we aim to study how the principles of melt alloying can be applied to 2D-HOIPs, and specifically, how the thermal and optical properties of 2D-HOIPs change after liquid-phase blending. The first, and probably the most critical, step is choosing the 2D-HOIPs which will undergo the melt alloying process. We select HOIPs based on a few criteria: (i) They need to have overlapping melt-processable windows,^40^ i.e., there should be a temperature range in which both melt but neither decomposes. (ii) They should differ in at least two out of the three structural components (organic cation, metallic cation, and halide). This way we ensure we will not study doping phenomena alone. (iii) They should have distinguishable thermal and optical properties (for example: they absorb light in different regimes of the electromagnetic spectrum), as this will enable us to easily detect changes in these properties upon mixing. With these in mind, we select (1-MHA)_2_PbI_4_ (1-MHA = 1-methylhexylammonium)—a liquid-forming visible range-absorbing 2D-HOIP,^37,47^ and (*S*-NEA)_2_PbBr_4_ (*S*-NEA = (*S*)-(−)-1-(1-naphthyl)ethylammonium)—a glass-forming near-UV-absorbing 2D-HOIP.^43,47^ These two candidates well-fulfill all three criteria mentioned above. We have synthesized these 2D-HOIPs in powder form, blended them in different ratios, and then studied how their properties change with the blend’s composition. Based on their thermal behavior, we could divide the blends into two groups: the first is the 1-MHA-rich samples, which melt upon heating and crystallize when cooled. The second group is the 1-MHA-poor samples, which form a glass after melting and recrystallize upon a second heating treatment. All of the samples were shown to be chemically homogeneous after melting, and maintained their homogeneity when recrystallized. As for their optical properties, a large difference in band gap was observed comparing the crystalline and the glassy samples. This difference was eliminated upon recrystallization, as the band gap continuously changes between the values of the pure materials.

## Results and Discussion

**Figure 1:**
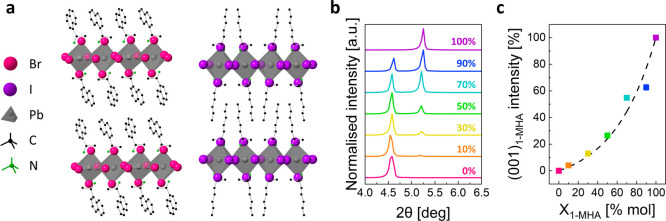
Structure of the physical mixtures: (a) crystal structures of (*S*-NEA)_2_PbBr_4_ (left) and (1-MHA)_2_PbI_4_ (right). The atoms are color-coded as follows: pink—Br; purple—I; gray—Pb; black—C; green—N. Hydrogen atoms were omitted for clarity. (b) XRD (001) reflections of the physical (as-is) blends (acquired using a wavelength of λ = 1.5406 Å). (c) Relative (001) reflection intensity of (1-MHA)_2_PbI_4_, calculated based on XRD peaks integration.

We synthesized (1-MHA)_2_PbI_4_ and (*S*-NEA)_2_PbBr_4_ based on previously reported procedures (see crystalline structures in Figure 1a).^37,43^ Then, we prepared physical mixtures of the two crystalline components in different ratios (see Experimental Section for full details). For clarity, from this point on, each sample will be named after the relative (molar) concentration of (1-MHA)_2_PbI_4_ (for example, the sample with *X*_1-MHA_ = 10% contains 10% mol of (1-MHA)_2_PbI_4_ and 90% mol of (*S*-NEA)_2_PbBr_4_, see Table S1). Consequently, the sample with *X*_1-MHA_ = 0% is pure (*S*-NEA)_2_PbBr_4_, while the *X*_1-MHA_ = 100% sample is pure (1-MHA)_2_PbI_4_. As can be seen from the (001) XRD reflections of the physical (as-is) blends in Figure 1b, all mixtures are crystalline. As the ratio of the 2D-HOIP components in each blend changes, the relative intensities of corresponding reflections change as well. To quantify this change, we fitted the (001) diffraction peaks of each phase to a Voigt function,^48^ and then calculated the relative intensity of the (1-MHA)_2_PbI_4_ diffraction peak. The results, presented in Figure 1c, indeed show a monotonic increase in relative intensity, which suggests a gradual change in blend composition.

**Figure 2:**
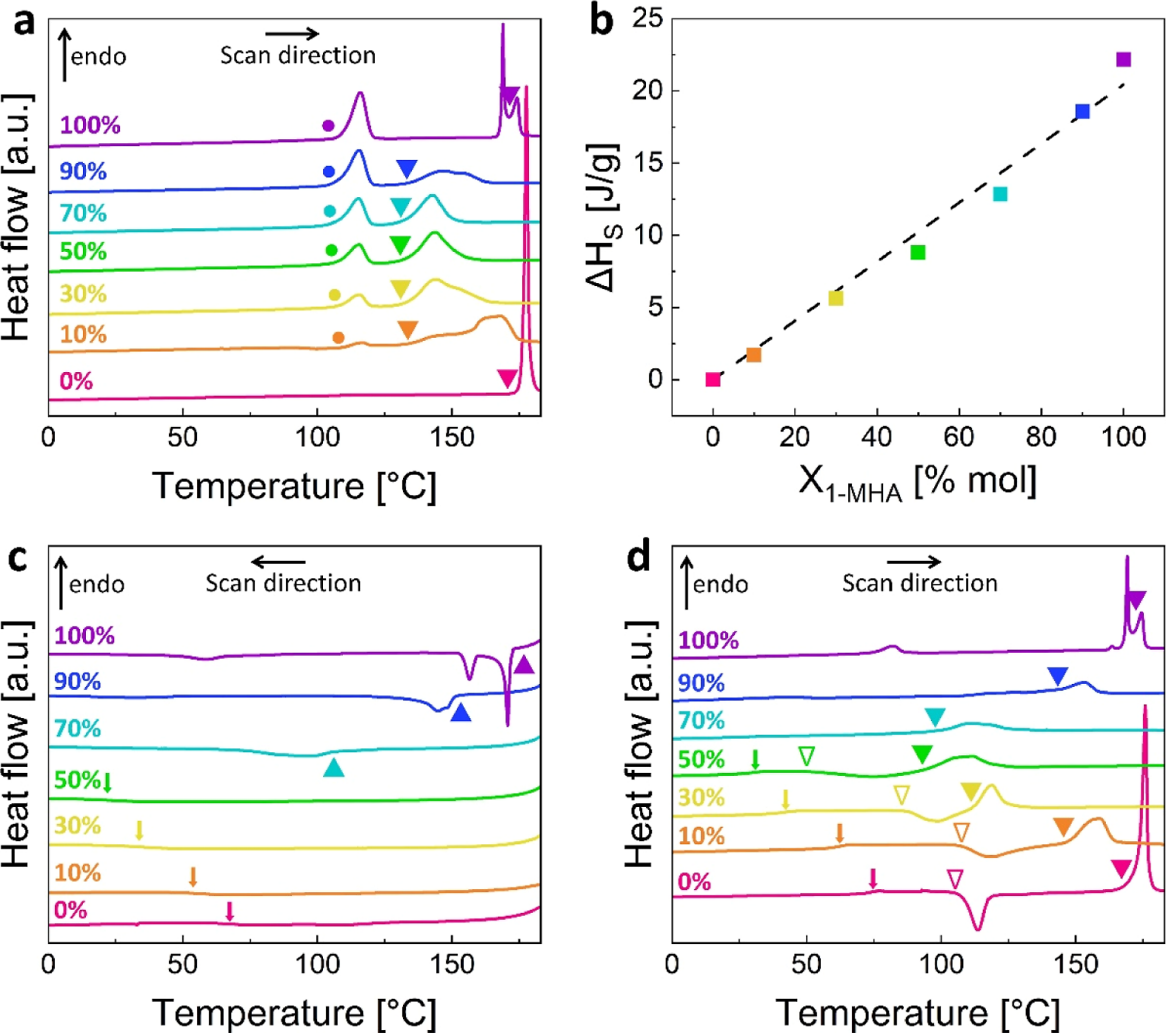
Thermal behavior of the blends during temperature changes: (a) DSC curves of the blends during the first heating step, from 0 to 185 °C. (b) Enthalpy of transformation for the (1-MHA)_2_PbI_4_ solid–solid transition. The dashed black line represents the fitted linear trend (*R*^2^ = 0.993) (c) DSC curves of the blends during the first cooling step, from 185 to 0 °C. (d) DSC curves of the blends during the second heating step, from 0 to 185 °C. All heating and cooling were performed at a rate of 10 °C min^–1^. The symbols represent as follows: full circles – (1-MHA)_2_PbI_4_ solid–solid transformation; downward arrows–glass transition; empty triangles–recrystallization onset; filled triangles–melting (facing down) and crystallization from a melt (facing up) onsets.

First, we investigate how the thermal properties of the 2D-HOIPs change upon blending. To this end, we used thermal gravimetric analysis (TGA) and differential scanning calorimetry (DSC). Upon heating, the DSC curves of the blends (Figure 2a) contain two main endothermic peaks. The first, around *T*_s_ = 115 °C (marked with full circles), is attributed to a solid–solid phase transformation of (1-MHA)_2_PbI_4_.^37^ This peak does not shift upon mixing with (*S*-NEA)_2_PbBr_4_. However, its intensity gradually increases with the concentration of (1-MHA)_2_PbI_4_ (*X*_1-MHA_). As a DSC peak integration represents the enthalpy of transformation, this is a direct measure of the (1-MHA)_2_PbI_4_ concentration in the blends. The highly linear (*R*^2^ = 0.993) trend presented in Figure 2b, suggests that the DSC measurements in this study well represent the blend (i.e., the blends were well-mixed).

The second peak in the DSC curves (down-facing filled triangles in Figure 2a) can be attributed to the melting of the 2D-HOIP blends. For *X*_1-MHA_ = 0% and *X*_1-MHA_ = 100% (i.e., the pure 2D-HOIPs) we measured melting temperatures of *T*_m_ = 176.7 °C and *T*_m_ = 172.8 °C, respectively. These values are in very good agreement with the previously reported melting points of these materials.^37,43^ Upon mixing, the melting point of the blends drops to ∼130 °C and stays roughly constant for all blending compositions (then rising back up for pure *X*_1-MHA_). This significant freezing point depression is evident for the well-performed blending and suggests a mutual effect of the HOIPs solely upon physical blending.

Next, we cooled down the samples back to 0 °C. Based on the DSC cooling curves in Figure 2c, the samples can be divided into two groups. The first group, consisting of 1-MHA-rich samples (*X*_1-MHA_ = 70%–100% mol, incl.), all crystallized upon cooling. However, the observed crystallization temperature differs from the melting temperature measured during the first heating scan, due to the liquid phase alloying. The crystallization temperatures of the alloys are indicated by the filled triangles in Figure 2c. This transformation between the crystalline (solid) phase and the melt (liquid) reoccurs at similar temperatures during the subsequent heating, as shown by the filled triangles in Figure 2d. This, together with the absence of any changes in the XRD pattern (Figure S2h–j) before and after melting, suggests that the melting process in these samples is reversible.

In contrast, the second group, comprising of 1-MHA-poor samples (0%–50% mol, incl.), did not exhibit crystallization upon cooling. Instead, these samples demonstrated a glass transition, occurring at a distinct temperature for each composition, as marked by down-facing arrows in Figure 2c. The reverse glass transition reoccurs upon heating (down-facing arrows in Figure 2d), followed by an exothermic recrystallization peak (empty triangles in Figure 2d). Upon further heating, the recrystallized solid melts once again, as indicated by the filled triangles in Figure 2d.

**Figure 3:**
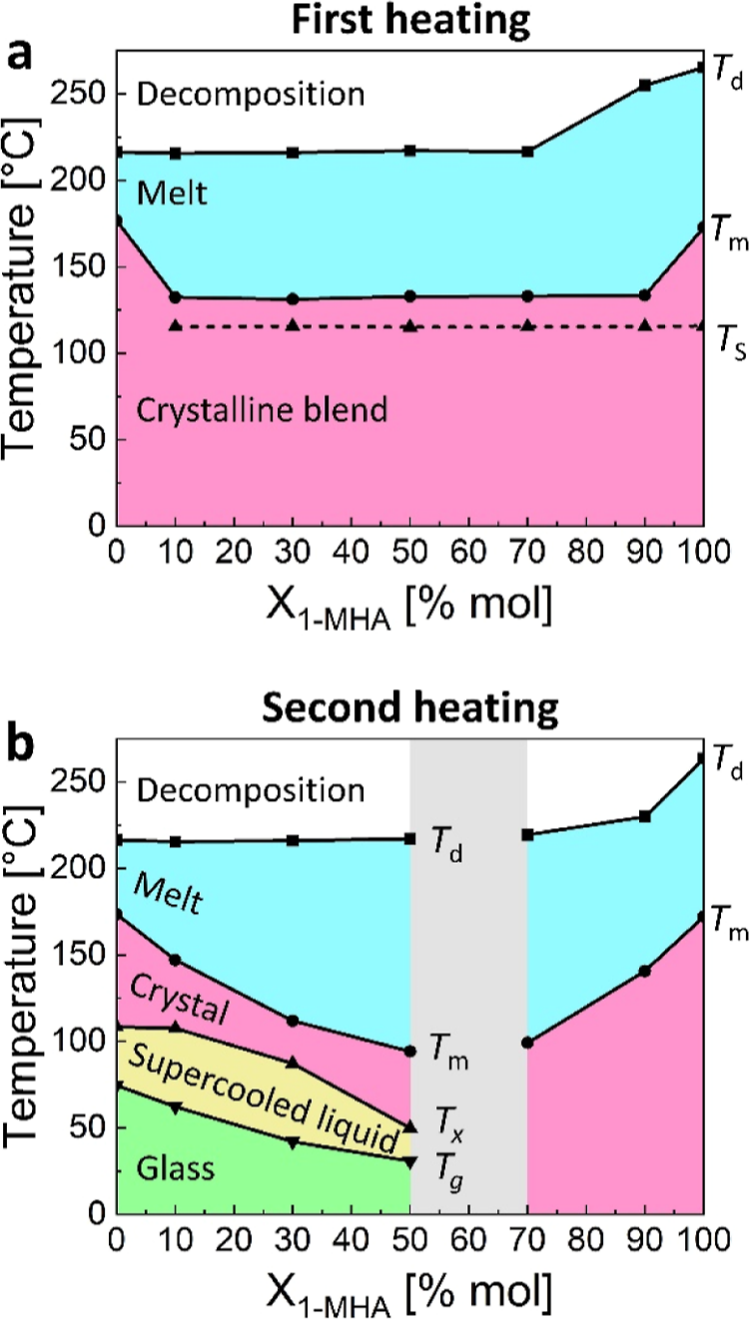
Thermal properties of the blends: The blends phase, as measured during the (a) first heating, and (b) second heating DSC steps (from 0 to 185 °C, at a rate of 10 °C min^–1^). Each black line represents a different thermal transition, as indicated by the mentioned temperatures: solid–solid transition of (1-MHA)_2_PbI_4_ (*T*_s_); melting (*T*_m_); decomposition (*T*_d_); glass transition (*T*_g_); and recrystallization (*T*_*x*_). The gray area in (b) represents the border between the glass-forming (left) and the liquid-forming (right) blends.

Combining values extracted from the DSC and the TGA measurements (see Figure S1 for TGA curves), we have constructed *T*–*X* diagrams for the studied 2D-HOIPs blends during the first and second heating DSC scans (see Figure 2). These plots, presented in Figure 3, represent the most stable state of the blends, based on their composition and temperature. During the first heating step, the blends can exist in one of three states (Figure 3a): a crystalline blend, a melt, or as decomposition products. However, during the second heating step (after melting and cooling, Figure 3b), in addition to their crystalline and liquid states, the blends can possess a glassy or a supercooled liquid structure. Note that following the liquid phase blending, the temperatures of all thermal transitions gradually change according to *X*_1-MHA_.

**Figure 4:**
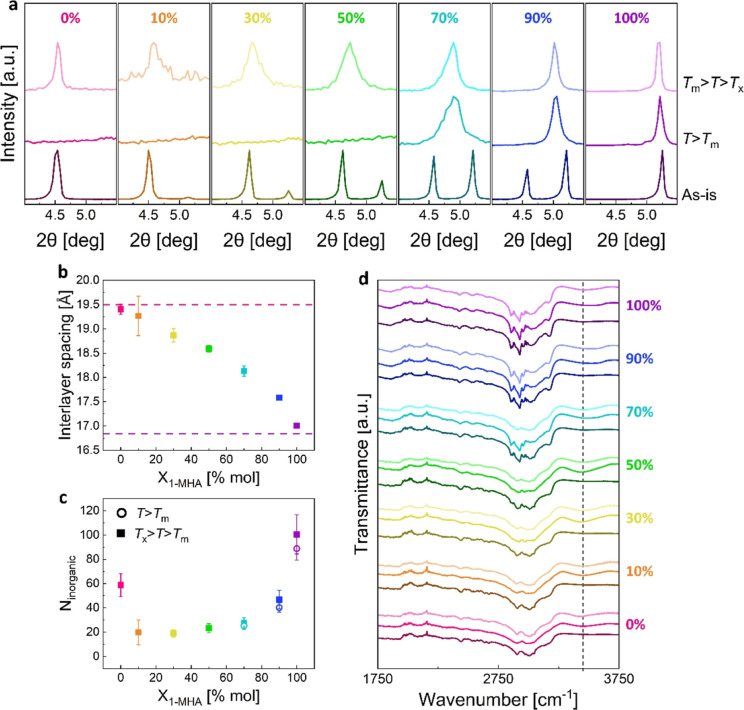
Structural analysis of the blends: (a) The (001) XRD peaks after different heating stages: bottom row—as-is blends; middle row—after ex situ melting at *T* > *T*_m_; top row—after ex situ recrystallization at *T*_m_ > *T* > *T*_*x*_. The data was acquired after cooling the samples back to room temperature. In each pair of diffraction peaks for the physical blends (bottom row), the left peak corresponds to pure (*S*-NEA)_2_PbBr_4_, while the right peak corresponds to pure (1-MHA)_2_PbI_4_. (b) Inorganic interlayer distance, and (c) average number of inorganic layers along the *c*-direction in a HOIP crystal, calculated based on the (001) XRD reflections. The dashed lines in (b) represent the values for the pure 2D-HOIPs, as measured using XRD before melting (as-is 0% and 100% samples). The open circles in (c) represent the number of inorganic layers after melting. (d) FTIR spectra of the blends. Each set of spectra corresponds to a different blend ratio. The stacking in each set is the same as in panel (a). The vertical dashed line represents the position of the free NH_3_ absorbance peak.

Following the thermal characterization, we were interested in how the structure of the 2D-HOIPs blends changes at different stages of the heating process. To this end, XRD measurements after two additional heat treatments were performed. First, we measured the samples using XRD after ex situ heating to 185 °C (i.e., after melting, *T* > *T*_m_). Then, we reheated the samples (ex situ, once again), each to a different temperature, which is lower than its *T*_m_ but higher than *T*_*x*_ (after recrystallization, *T*_m_ > *T* > *T*_*x*_, see Experimental Section and Table S1 for more details). The (001) reflections of the blends, after each thermal treatment, are presented in Figure 4a (as the full diffractograms are presented in Figure S2). The XRD (middle row in Figure 4a) results of the blends after melting corroborate the DSC findings: While the 1-MHA-rich samples return to their crystalline form, the 1-MHA-poor ones do not crystallize upon cooling, and instead form an amorphous (glassy) phase.

When comparing the diffraction patterns of the as-is blends and the corresponding recrystallized blends, the first noticeable change is the merging of diffraction peaks. Each as-is blend possesses two (001) reflections, one for each 2D-HOIP (with their ratio changes according to composition, as discussed earlier). However, the recrystallized blends (and the 1-MHA-rich blends after melting) each have one (001) reflection. The merged diffraction peak is positioned between the two original peaks, and shifts toward higher 2θ values with increasing (1-MHA)_2_PbI_4_ concentration. This is the first evidence of the homogeneity of the recrystallized samples, as they seem to adopt a structure which is an average of the original 2D-HOIPs structures.

By fitting the recrystallized blends (001) reflections to a Voigt function,^48^ combined with Bragg law,^49^ and the previously reported structures of the 2D-HOIPs,^37,43^ we can calculate the average distance between inorganic adjacent layers in the 2D-HOIP
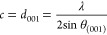
1where λ is the X-ray wavelength (1.5406 Å in this case), and θ_(001)_ is the Bragg angle of the (001) diffraction peak. As presented in Figure 4b, the interlayer spacing gradually changes between the values of pure (*S*-NEA)_2_PbBr_4_ and pure (1-MHA)_2_PbI_4_. We note that the interlayer distance slightly changes after heating, even for the pure samples, though the change is within experimental error for (*S*-NEA)_2_PbBr_4_. For pure (1-MHA)_2_PbI_4_ the interlayer distance increases after heating, which may imply a minor rearrangement of the alkyl chains or stress relaxation.^50,51^ Note that while the *S*-NEA cation was shown to be able to form one-dimensional (1D) perovskite structures in the presence of iodide,^52^ we did not observe evidence for the formation of the 1D polymorph in the XRD of our samples.

Another difference between the (001) reflections is their full-width half-maximum (fwhm) values. This value can be affiliated with the crystallinity of the samples, as more crystalline samples are expected to demonstrate narrower Bragg peaks.^49^ Based on the Lorentzian portion of the (001) reflection fwhm (*W*_L_, as fitted), we can approximate the number of inorganic layers, according to eq 2
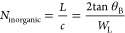
2where L is the coherence length (“average crystallite size”) along the *c*-direction.^48^ After recrystallization, the coherence length of the blends is significantly lower compared to the pure 2D-HOIPs (Figure 4c). While the crystal structure of the nonglass-forming 1-MHA-rich samples does not change after the second heating treatment (Figure S2h–j), we note an increase in their particle size (see empty circles in Figure 4c). This change is due to thermal-induced crystal growth. To confirm that the observed changes in the position and shape of the diffraction peaks indeed originate from structural changes, rather than instrumental effects, we repeated our analysis for the (002) reflections (the second most intense diffraction peaks after melting and recrystallization, located at 2θ = 9–10°, see Figure S2). The obtained values, both the distance between inorganic layers and their number, are in excellent agreement with the ones obtained for the (001) reflection (see comparison in Figure S3). Hence, we can confirm the accuracy of the structural analysis.

While XRD provides information mainly about the inorganic parts in the crystal, we were also interested in the blending-induced changes that occur in the organic component. To this end, we used Fourier transform infrared (FTIR) spectroscopy. For each blend, (*X*_1-MHA_ value) the FTIR spectra, acquired after the different heating stages (as-is, after melting, and after recrystallization), are profoundly similar (see Figure S4a). This suggests no decomposition of the organic components, as was also corroborated using TGA (Figure S1). One existing difference between the spectra is the appearance of the broad absorbance peak at ∼3500 cm^–1^ after melting, which is absent in the physical (as-is) blends (marked with a vertical dashed line in Figure 4d, and zoomed-in in Figure S4b). This peak corresponds to free amino (NH_3_^+^) groups; as such, its appearance represents the thermal-induced breaking of the hydrogen bonds between the organic cation and the inorganic layers.^53−56^ As expected, this peak is the most intense in the glassy samples (*X*_1-MHA_ ≤ 50% mol, see Figure S4b). Surprisingly, it is clearly visible after melting in all the samples, including the nonglass forming ones (*X*_1-MHA_ ≥ 70% mol). This means that the loss of organic–inorganic interaction (i.e., breaking of hydrogen bonds) occurs upon melting, and is not necessarily only due to glass formation. Moreover, Figures 4d and S4b undoubtedly demonstrate that this peak still exists in all the blends after recrystallization, although at a weaker intensity, especially for the glass-forming samples. This might suggest a partial reformation of the hydrogen bond upon recrystallization of the glass.

**Figure 5:**
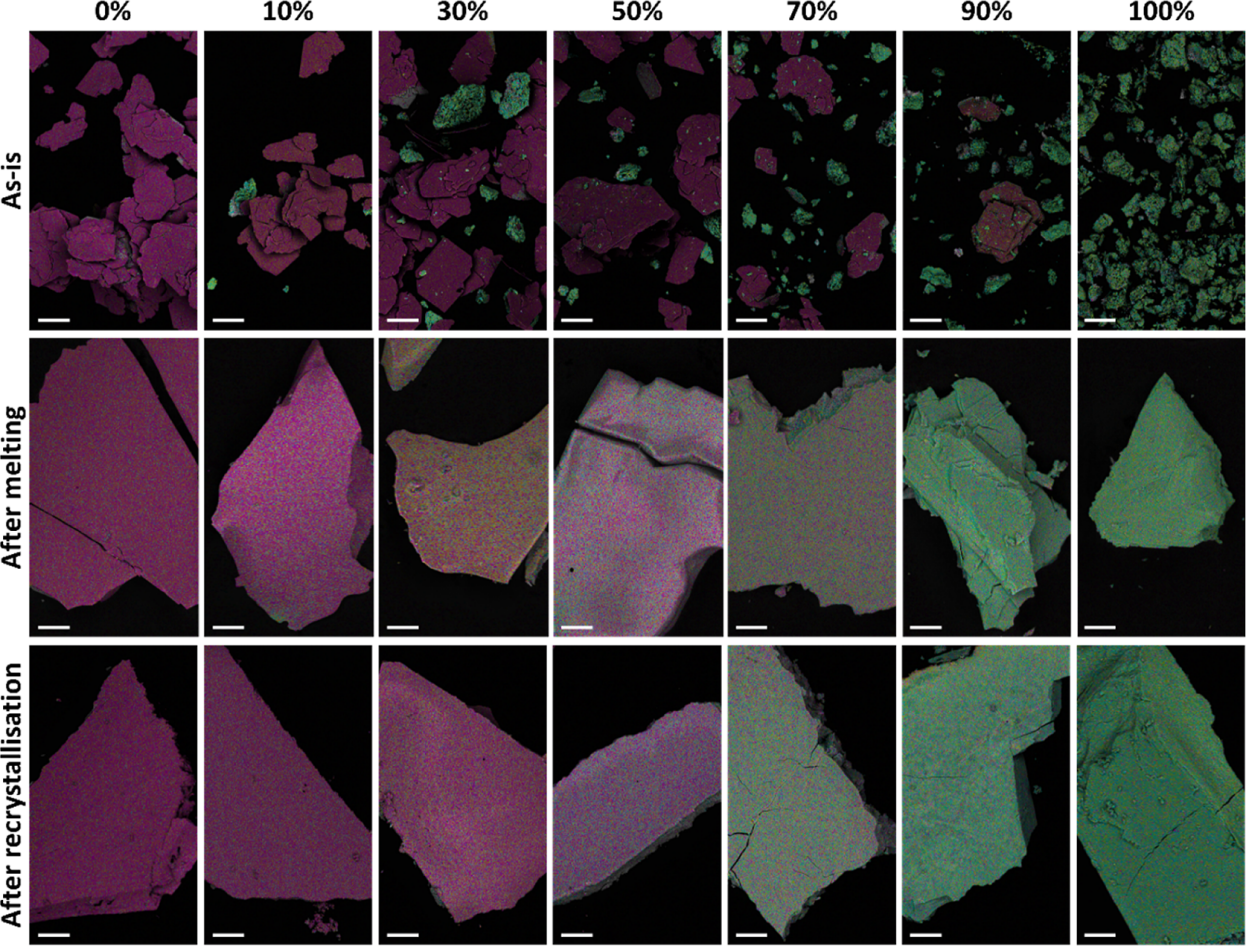
Chemical analysis of the blends: EDS elemental maps, presented on top of the corresponding backscattered electron micrographs, of the samples at different stages: as-is (top row), after melting (middle row), and after recrystallization (bottom row). The colors of each element are as follows: Pb—yellow; Br—magenta; I—cyan. Scalebars: 100 μm.

The merge of the two (001)_*S*-NEA_ and (001)_1-MHA_ XRD peaks to a single (001)_blend_ reflection (Figure 4a), together with the gradual decrease in interlayer spacing (Figure 4b), suggests that the recrystallized form is chemically homogeneous, i.e., Br and I (and also, *S*-NEA and 1-MHA) are evenly distributed in the crystal. To corroborate this conclusion, we employed energy-dispersive X-ray spectroscopy (EDS) in the scanning electrons microscope (SEM) and constructed elemental maps for each sample (Figure 5).

The top row in Figure 5 presents the elemental maps of the as-is blends. The 0% sample on the left contains only purple plate-like crystals, as this sample is pure (*S*-NEA)_2_PbBr_4_. On the right side, the 100% sample consists solely of green aggregates, which correspond to the pure (1-MHA)_2_PbI_4_. Between them, the physical mixtures contain crystals of both types, as the green crystals seem to increase in relative amount as the composition of the blend increases. Note that due to the significant difference in the morphology of the crystals, they can be easily distinguished even using the secondary electrons (SE) signal in the SEM (see Figure S5).

On the middle row in Figure 5, the elemental maps of the blends after melting are shown. First, it is clear that the grains are much bigger, due to the melting process. Moreover, it seems that the 1-MHA-poor samples (*X*_1-MHA_ ≤ 50% mol), possess more curved edges, when compared to the sharp edges of the 1-MHA-rich samples (*X*_1-MHA_ ≥ 70% mol, see also Figure S5). This difference further confirms the glass formation abilities of the first group. As for the halide distribution, in all the samples, the elemental maps appear to be completely uniform, i.e., the halides are evenly distributed in the solid. This was further confirmed by using higher magnifications, as presented in Figure S6 (top row). The hue of the crystals gradually changes from purple to green between each sample, which indicates a graduate decrease in Br concentration, and a corresponding increase in I concentration. Quantifying the relative amounts of Pb, Br, I in each sample reveals that the concentration of each element in the blend after melting is precisely determined by the stoichiometry of the 2D-HOIPs and their relative amount in the blend (see Figure S7a,b).

The bottom row in Figure 5 presents the maps of the blends after recrystallization. Here, all samples have sharp edges, as expected due to their crystallinity (see also bottom row in Figure S5). Once again, the hue of the crystals gradually changes, as dictated by the gradual increase in the relative I concentration (Figure S7, bottom row). Furthermore, it seems that the samples remain homogeneous upon recrystallization, as no fluctuations in halide concentration were observed (not even at high magnification, see Figure S6, bottom row).

**Figure 6:**
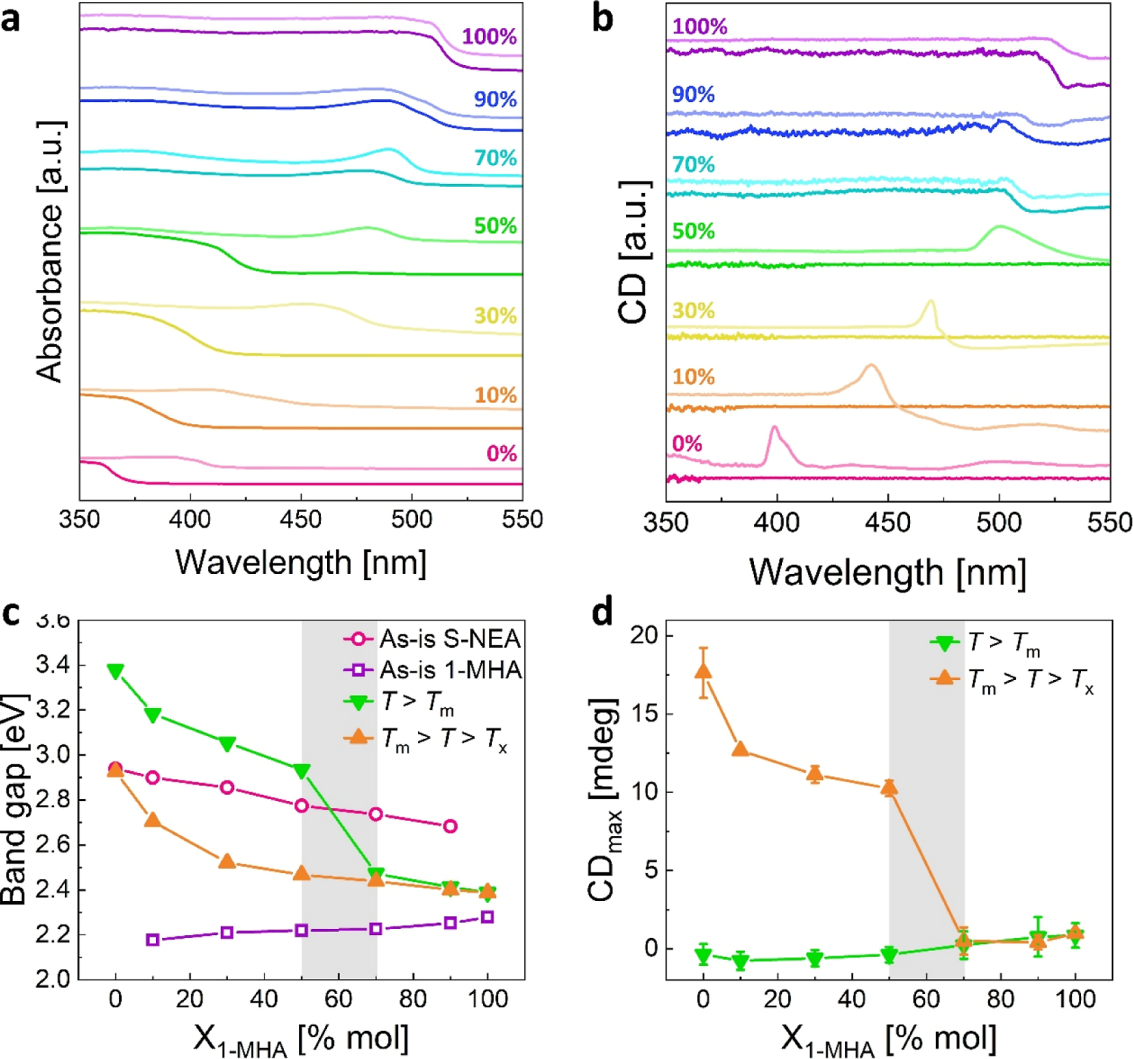
Optical properties of the blends: (a) UV–vis absorbance spectra and (b) CD spectra of the samples. In each pair, the bottom (dark) curve represents the spectrum recorded after melting, and the top (light) curve represents the spectrum after recrystallization. (c) The optical band gap(s) of each sample, as calculated using the Tauc method. Empty data points represent diffuse reflectance-based data, and full points represent absorbance-based data. (d) Maximal CD response calculated based on the difference between the CD peak maxima and the average value of low-wavelength plateau. The gray areas represent the border between glass-forming (1-MHA-poor) and liquid-forming (1-MHA-rich) samples.

Lastly, to study how the blend-induced changes affect the physical properties of the HOIPs, we measured their optical properties using ultraviolet and visible range (UV–vis) spectroscopy. For the samples after melting and recrystallization, the perovskite layer was sufficient for a direct measure of the absorbance spectra (see Experimental Section for sample preparation details and Figure 6a for spectra). However, due to the scattering nature of the powdered as-is samples, we measured their diffuse reflectance and employed the Kubelka–Munk method (see Figure S8).^57,58^

The optical band gap(s) of each sample, calculated based on their absorbance using the Tauc method (see Figure S9),^52,57,58^ is presented in Figure 6c. The empty data points (pink circles and purple squares) represent the band gap(s) of the as-is powders, before melting. For each blend (10% ≤ *X*_1-MHA_ ≤ 90%), two absorbance onsets are present, corresponding to two band gaps–one for each HOIP. However, the two semiconductors affect each other’s optical properties solely upon physical blending, as the band gaps gradually change with the blend composition. This mutual effect, occurring only due to physical blending, may result from halide exchange–a rapid halide diffusion known to occur in HOIPs (see EDS elemental maps in Figure S10).^59,60^

The band gap of the blends after melting is represented by green down-facing triangles in Figure 6c. Here, each sample has one band gap, which further confirms the melting-induced homogeneity discussed previously. The band gaps of the samples gradually decrease with the increase in *X*_1-MHA_, however, this decrease is not continuous–the band gap sharply drops in value between *X*_1-MHA_ = 50% mol and 70% mol. As was shown using DSC (Figure 2) and XRD (Figure 4), 1-MHA-poor samples possess a glassy (amorphous) structure after melting, hence their band gap is significantly higher.^61,62^ 1-MHA-rich samples, which crystallize after melting, have a lower band gap corresponding to their crystalline phase. While one would expect no change in the band gap of pure (1-MHA)_2_PbI_4_ due to melting, this is not the case. The slight increase in the band gap of pure (1-MHA)_2_PbI_4_, as shown in Figure 6, suggests a slight change in the structure of the HOIP. This corroborates the small increase in the interlayer spacing observed using XRD and the melt-induced hydrogen bond breaking observed using FTIR (Figure 4).

The orange up-facing triangles in Figure 6c represent the band gap of the samples after heating to *T*_m_ > *T* > *T*_*x*_. For the 1-MHA-poor samples, thermal-induced recrystallization results in a significant reduction of the band gap, of about 0.5 eV. In contrast, the band gap of the 1-MHA-rich samples shows no significant change between the two heating stages. This is expected as the only difference in structure between the two heating stages is a slight increase in crystallite size, as observed in the XRD (Figure 4c). Now, as the samples are all crystalline, their band gap continuously changes between those of pure (*S*-NEA)_2_PbBr_4_ and (1-MHA)_2_PbI_4_. Note that the band gap of pure (*S*-NEA)_2_PbBr_4_ (*X*_1-MHA_ = 0% mol) is identical for the as-is and the recrystallized sample, and in excellent agreement with previous studies.^52^ This is a further confirmation of the reversibility of the glass formation of this 2D-HOIP.^43^

As one of the blended 2D-HOIPs contains chiral molecules (*S*-NEA), we investigated the response of the blends to polarized light. To this end, we measured the circular dichroism (CD) spectra of the blends after melting, and once again after recrystallization. All CD spectra are presented in Figure 6b (and Figure S11). No significant CD response was recorded for the glassy samples, as their CD spectra are completely featureless. Due to the melt-induced hydrogen bonds breaking, no chirality transfer is expected between the organic and the inorganic layers.^52,54^ Surprisingly, the nonglass forming samples (*X*_1-MHA_ ≥ 70% mol) do demonstrate some nonzero CD at high wavelengths, including the pure (1-MHA)_2_PbI_4_, which does not contain any chiral molecules. Since no optical absorption was recorded in this region (Figure 6a), and due to the high scattering nature of the crystalline samples, we attribute this signal to an artifact.^63,64^ This scattering is also the reason we were unable to record the CD spectra for the as-is physical blends.

After recrystallization, a characteristic peak in the CD spectra of pure (*S*-NEA)_2_PbBr_4_ appears. This peak is associated with the dipole-induced chirality transfer between the organic and inorganic sublayers of the crystal.^52^ Our CD spectrum is in good agreement with the one presented by Jana et al.,^52^ who studied the chirality transfer in (*S*-NEA)_2_PbBr_4_. As presented in Figure 6b, all the recrystallized 1-MHA-poor samples demonstrate CD response in the form of a single peak. The position of the peak shifts toward higher wavelengths, in accordance with the redshift of the samples’ band gap (Figure 6c). Moreover, the peak’s maximal intensity, corresponding to the strength of chiral absorption,^65^ decreases with the decrease in the concentration of the chiral molecules in the blend (see Figure 6d). Note that as the light scattering from the recrystallized samples is much more intense compared to the glassy ones, these recrystallized samples also demonstrate a nonzero CD response at the high, nonoptically active, wavelength region.

The second heat treatment does not affect the CD response of the 1-MHA-rich samples, with no changes in their CD spectra (Figure 6b). Notably, no CD peaks were observed, even with the presence of chiral *S*-NEA molecules in the blend. This outcome may be attributed to an insufficient concentration of *S*-NEA in these samples, which hinders both chirality transfer and glass formation.

## Conclusions

This work demonstrates, for the first time, how the principles of melt alloying can be applied to hybrid organic–inorganic perovskite blends. The two 2D-HOIPs of choice, (*S*-NEA)_2_PbBr_4_ and (1-MHA)_2_PbI_4_ (a glass-forming and a liquid-forming 2D-HOIPs, respectively), were synthesized in their crystalline form, blended in different ratios, and underwent mutual melting. This allowed us to construct *T*–*X* diagrams for this 2D-HOIP pair and show how their thermal properties can be tuned upon blending. Specifically, we have demonstrated that the melting temperature of the blends drops upon physical mixing, from >170 °C (for the pure 2D-HOIPs) to ∼130 °C. It then changes once more when a second melting is performed. The thermal behavior of the blends allows us to divide the different compositions into two groups: 1-MHA-poor blends, which form a glassy state upon cooling from a melt, and 1-MHA-rich blends, which crystallize back to their thermodynamically stable structure. The first group, which includes blends with up to 50% mol of the nonglass-forming 2D-HOIP, possesses a glass transition behavior, at temperatures determined by the composition of the blend.

We then employed XRD to explore the homogeneity of the blends. The two different (001) reflections, which can be clearly resolved in the as-is physical mixtures, merge to one diffraction peak upon melting and recrystallization. This merge of XRD reflections, together with the gradual change in the distance between the inorganic layers, suggests the formation of a homogeneous crystalline blend (however, with poor crystallinity compared to the as-is blend). Such homogeneity, as was further confirmed by elemental analysis using SEM–EDS, stands in contrast to the previously shown domain structure formed by liquid blending of MOFs.^27^

Lastly, we have monitored how the optical properties of the samples change due to melting, glass formation and recrystallization, depending on the blend’s composition. After melting, the glassy samples had a significantly higher band gap than the crystalline ones, as was measured using UV–vis optical absorption. In each of these groups, the band gap was gradually changing according to the blend’s composition. After a second heat treatment, the recrystallization of the first group induced a drop in their band gap, while it did not change the band gap of the crystalline blends. Upon measuring the response of the glassy samples to circularly polarized light, no chirality transfer was observed, as indicated by their featureless CD spectra. This lack of transfer is attributed to the disruption of organic–inorganic hydrogen bonds, as shown by FTIR. Following recrystallization, these bonds were partially reformed, leading to a characteristic CD response, evident as a peak in the CD spectra of these samples. The intensity of this peak was found to correlate with the concentration of chiral molecules within the blend. However, further work will be needed to better understand the nature and origin of the CD signals, or the lack of them, including a more quantitative analysis.

Overall, this work demonstrates how classical melt-based processing methods can be applied to novel meltable hybrid perovskite binary systems. It further emphasizes the importance of understanding the factors governing HOIPs melting, as it provides a new approach for properties tuning of the perovskite using a solvent-free method.

## Experimental Section

(1-MHA)_2_PbI_4_ synthesis: 2.765 g of lead iodide (PbI_2_, 99%, Aldrich) were dissolved in 50 mL concentrated hydroiodic acid (HI stabilized, 57% at, Thermo Scientific), in a 100 mL conical flask. When the dissolution was complete, 1.805 mL of 1-methylhexylamine (1-MHA, 2-heptylamine, ≥98%, Thermo Scientific) was added to the solution during stirring. At this stage, precipitation occurred. After a few minutes of stirring, the flask was sealed and transferred to a silicone oil bath, which was placed on a hot plate and heated to 90 °C, and the solution was stirred until complete dissolution was achieved once again. Then, the hot plate was turned off and the solution was left to naturally cool down to room temperature overnight, as the stirring was left on. Cooling down while stirring resulted in the precipitation of micron-size deep-yellow powder. The flask content was transferred to a Falkon tube. The tube was centrifuged, and the acidic solute was disposed of. Then, hexane (Fisher Scientific) was added to the tube, and it was vigorously shaken, centrifuged, and the hexane was disposed of. This process was repeated 3 times. Finally, the tube was left for a final drying stage under vacuum, at room temperature, overnight.

(*S*-NEA)_2_PbBr_4_ synthesis: 1.350 g of lead bromide (PbBr_2_, ≥98%, Sigma-Aldrich) were dissolved in a mixture of 15 mL concentrated hydrobromic acid (HBr, 48% wt, Thermo Scientific) and 35 mL of deionized water (DIW), in a 100 mL conical flask. When the dissolution was complete, 1.17 mL of (*S*)-(−)-1-(1-naphthyl)ethylamine (*S*-NEA, 99%, Thermo Scientific) was added to the solution during stirring. At this stage, some precipitation occurred. After a few minutes of stirring, the flask was sealed and transferred to a silicone oil bath, which was placed on a hot plate and heated to 90 °C, and the solution was stirred until complete dissolution was achieved once again. Then, the hot plate was turned off and the solution was left to naturally cool down to room temperature overnight, as the stirring was left on. Cooling down while stirring resulted in the precipitation of micron-size pale-yellow powder. The flask content was transferred to a Falkon tube. The tube was centrifuged and the acidic solute was disposed of. Then, diethyl ether (Et_2_O, ≥99.8%, Sigma-Aldrich) was added to the tube, and it was vigorously shaken, centrifuged, and the Et_2_O was disposed of. This process was repeated 3 times. Finally, the tube was left for a final drying stage under vacuum, at room temperature, overnight.

Blends preparation: 2D-HOIPs blends were prepared by weighing the appropriate amount of each perovskite powder (see Table S1). After weighing, the powders were transferred to glass tubes, sealed, and left overnight on a rotating tube shaker.

Thermal gravimetric analysis: TGA was performed in a simultaneous thermal analyzer (SDT 650, TA Instruments). A few mg of each blend was transferred to an alumina crucible, and scanned from room temperature to 700 °C, at a rate of 10 °C min^–1^, under an inert Ar atmosphere.

Differential scanning calorimetry: DSC measurements were performed using a DSC 2500 instrument (TA Instruments). A few mg of each blend was transferred into an aluminum pan, followed by sealing the pan. Each sample was stabilized at 0 °C, then heated to 185 °C and cooled back down to 0 °C, twice, at a rate of 10 °C min^–1^. All scans were performed under an inert N_2_ atmosphere.

X-ray diffraction: XRD was performed in a Bruker D8 Advance diffractometer (Cu–Kα source, λ = 1.5406 Å). Each sample was mounted on a low background Si holder (5 mm in diameter) and scanned in a 2θ range of 3°–40° (scan rate: 2° min^–1^).

Ex situ heating XRD: Heating the samples was done in the SDT. To study the structure after melting, all of the samples were heated to 185 °C. the samples were heated at a rate of 20 °C min^–1^, then kept at this temperature for 3 min, and cooled down to room temperature at 30 °C min^–1^. The samples were taken out of the SDT pans, transferred to XRD holders, and scanned. To study the structure after recrystallization, a second batch of samples underwent the same heating cycle, followed by a second cycle: heating (20 °C min^–1^) to ∼15 °C below each sample’s *T*_m_, isotherm of 15 min, then cooling at 30 °C min^–1^. For each sample, we made sure that the selected final temperature is higher than the recrystallization temperature (Table S1). Once again, the samples were transferred to XRD holders and scanned.

Fourier transform infrared spectroscopy: Spectra were acquired using a ThermoFisher Scientific Nicolet iS50 FTIR spectrometer. A few mg of each sample (as-is, after melting and after recrystallization, where melting and recrystallization were performed in the SDT) were placed in the spectrometer sample holder and scanned in the range of 500–4000 cm^–1^.

Scanning electron microscopy: Samples were melted and recrystallized in the SDT, then spread on a conductive carbon tape which was adhered to an SEM stainless steel stub. Micrographs were acquired using the secondary electrons detector in a Zeiss Gemini SEM. The energy of the primary was set to 1.5 keV.

Energy dispersive X-ray spectroscopy: EDS maps were measured in the Zeiss Gemini SEM. Electron images were taken using the backscattered detector. The energy of the primary electrons was set to 15 keV. Elemental maps and quantification were done in the Oxford Aztech software.

Optical absorption: Microscope glass slides were cut to dimensions of ∼2 × 2 cm. The slides were submerged in absolute ethanol (EtOH, ≥99.8%, Fisher Scientific), sonicated for 30 min, and then thoroughly washed with EtOH. This cleaning process was repeated once with Acetone (≥99%, Sigma-Aldrich) and once more with iso-propanol (iPrOH, 99.6%, Thermo Scientific). Finally, the clean slides were placed on a lint-free paper, and dried in air overnight. To prepare the samples, about 100 mg of each powder bland was placed on a clean glass slide, and then covered with a second clean slide. The slides were sandwiched between preheated stainless-steel (SS) plates, and placed on a preheated heating stage at 185 ± 2 °C. On top of the SS plate, we placed an SS weight, to press the glass slide together. After 10 min, the samples were taken off the heating stage and were left to naturally cool down. The samples were transferred to a spectrophotometer (Cary 60), and their absorbance was recorded in a wavelength range of 350 to 800 nm. Two clean glass slides, with no sample, were used as blank. Then, we repeated the hating and measurement once again, this time by heating each sample to a different temperature, which was ∼15 °C below its melting temperature (see Table S1). The band gap of the samples was calculated using the Tauc method.^57,58^

Diffuse reflectance measurements: Around 10 mg of each sample blend was sandwiched between two clean glass slides. Due to the scattering nature of the powders, their reflectance (%R) was measured using a PerkinElmer Lambda 750 spectrophotometer, equipped with an integration sphere. Then, their absorbance was calculated using the Kubelka–Munk equation, and the band gap was determined as described above.^57,58^

Circular dichroism: CD spectra were measured for the blends after melting and recrystallization (sample preparation was carried out in the same way as for optical absorption measurments). All spectra were acquired using a Jasco J-1500 CD Spectrometer with 50 nm/min scan speed and 0.1 nm data pitch. For noise reduction, each sample was scanned twice, and the two spectra were accumulated.

## Supporting Information Available

Additional experimental details, including TGA, XRD, FTIR, SEM, EDS, optical absorption, and CD results (PDF)

## References

[ref1] Davis J. R.; Davis J. R. Alloying: Understanding the Basics; ASM International: Materials Park, United States, 2001.

[ref2] Anantharaman T. R.; Suryanarayana C. Review: A Decade of Quenching from the Melt. J. Mater. Sci. 1971, 6 (8), 1111–1135. 10.1007/BF00980610.

[ref3] Sohn H. Y.; Sridhar S. Descriptions of High-Temperature Metallurgical Processes. In Fundamentals of Metallurgy; Elsevier, 2005; pp 3–37.

[ref4] Cui X.; Cui H.; Wu Y.; Liu X. The Improvement of Electrical Conductivity of Hypoeutectic Al-Si Alloys Achieved by Composite Melt Treatment. J. Alloys Compd. 2019, 788, 1322–1328. 10.1016/j.jallcom.2019.02.242.

[ref5] Narayan Prabhu K.; Ravishankar B. N. Effect of Modification Melt Treatment on Casting/Chill Interfacial Heat Transfer and Electrical Conductivity of Al–13% Si Alloy. Mater. Sci. Eng., A 2003, 360 (1–2), 293–298. 10.1016/S0921-5093(03)00467-2.

[ref6] Manasijević D.; Balanović L.; Marković I.; Gorgievski M.; Stamenković U.; Božinović K. Microstructure, Melting Behavior and Thermal Conductivity of the Sn–Zn Alloys. Thermochim. Acta 2021, 702, 178978 10.1016/j.tca.2021.178978.

[ref7] Deev V.; Prusov E.; Ri E.; Prihodko O.; Smetanyuk S.; Chen X.; Konovalov S. Effect of Melt Overheating on Structure and Mechanical Properties of Al-Mg-Si Cast Alloy. Metals 2021, 11 (9), 1353 10.3390/met11091353.

[ref8] Nikanorov S. P.; Volkov M. P.; Gurin V. N.; Burenkov Yu. A.; Derkachenko L. I.; Kardashev B. K.; Regel L. L.; Wilcox W. R. Structural and Mechanical Properties of Al–Si Alloys Obtained by Fast Cooling of a Levitated Melt. Mater. Sci. Eng., A 2005, 390 (1–2), 63–69. 10.1016/j.msea.2004.07.037.

[ref9] Aversa A.; Lorusso M.; Cattano G.; Manfredi D.; Calignano F.; Ambrosio E. P.; Biamino S.; Fino P.; Lombardi M.; Pavese M. A Study of the Microstructure and the Mechanical Properties of an Al Si Ni Alloy Produced via Selective Laser Melting. J. Alloys Compd. 2017, 695, 1470–1478. 10.1016/j.jallcom.2016.10.285.

[ref10] Cao S.; Chen Z.; Lim C. V. S.; Yang K.; Jia Q.; Jarvis T.; Tomus D.; Wu X. Defect Microstructure, and Mechanical Property of Ti-6Al-4V Alloy Fabricated by High-Power Selective Laser Melting. JOM 2017, 69 (12), 2684–2692. 10.1007/s11837-017-2581-6.

[ref11] Correlo V. M.; Boesel L. F.; Bhattacharya M.; Mano J. F.; Neves N. M.; Reis R. L. Properties of Melt Processed Chitosan and Aliphatic Polyester Blends. Mater. Sci. Eng., A 2005, 403 (1–2), 57–68. 10.1016/j.msea.2005.04.055.

[ref12] Macosko C. W.; Guégan P.; Khandpur A. K.; Nakayama A.; Marechal P.; Inoue T. Compatibilizers for Melt Blending: Premade Block Copolymers. Macromolecules 1996, 29 (17), 5590–5598. 10.1021/ma9602482.

[ref13] Pilati F.; Marianucci E.; Berti C. Study of the Reactions Occurring during Melt Mixing of Poly(Ethylene Terephthalate) and Polycarbonate. J. Appl. Polym. Sci. 1985, 30 (3), 1267–1275. 10.1002/app.1985.070300330.

[ref14] Crist B.; Hill M. J. Recent Developments in Phase Separation of Polyolefin Melt Blends. J. Polym. Sci. B Polym. Phys. 1997, 35 (14), 2329–2353. 10.1002/(SICI)1099-0488(199710)35:14<2329::AID-POLB12>3.0.CO;2-E.

[ref15] Li Y.; Shimizu H. Toughening of Polylactide by Melt Blending with a Biodegradable Poly(Ether)Urethane Elastomer. Macromol. Biosci. 2007, 7 (7), 921–928. 10.1002/mabi.200700027. 17578835

[ref16] Jaratrotkamjorn R.; Khaokong C.; Tanrattanakul V. Toughness Enhancement of Poly(Lactic Acid) by Melt Blending with Natural Rubber. J. Appl. Polym. Sci. 2012, 124 (6), 5027–5036. 10.1002/app.35617.

[ref17] Anderson K. S.; Lim S. H.; Hillmyer M. A. Toughening of Polylactide by Melt Blending with Linear Low-density Polyethylene. J. Appl. Polym. Sci. 2003, 89 (14), 3757–3768. 10.1002/app.12462.

[ref18] Plouzeau M.; Piogé S.; Peilleron F.; Fontaine L.; Pascual S. Polymer/Dye Blends: Preparation and Optical Performance: A Short Review. J. Appl. Polym. Sci. 2022, 139 (36), e52861 10.1002/app.52861.

[ref19] Tsuji H.; Ikada Y. Crystallization from the Melt of Poly(Lactide)s with Different Optical Purities and Their Blends. Macromol. Chem. Phys. 1996, 197 (10), 3483–3499. 10.1002/macp.1996.021971033.

[ref20] Kelarakis A.; Yoon K. Optical Transparency in a Polymer Blend Induced by Clay Nanofillers. Eur. Polym. J. 2008, 44 (12), 3941–3945. 10.1016/j.eurpolymj.2008.08.030.

[ref21] Palza H.; Vergara R.; Zapata P. Composites of Polypropylene Melt Blended with Synthesized Silica Nanoparticles. Compos. Sci. Technol. 2011, 71 (4), 535–540. 10.1016/j.compscitech.2011.01.002.

[ref22] Li S.; Lin M. M.; Toprak M. S.; Kim D. K.; Muhammed M. Nanocomposites of Polymer and Inorganic Nanoparticles for Optical and Magnetic Applications. Nano Rev. 2010, 1 (1), 5214 10.3402/NANO.V1I0.5214. PMC3215211 22110855

[ref23] Wilson J. L.; Poddar P.; Frey N. A.; Srikanth H.; Mohomed K.; Harmon J. P.; Kotha S.; Wachsmuth J. Synthesis and Magnetic Properties of Polymer Nanocomposites with Embedded Iron Nanoparticles. J. Appl. Phys. 2004, 95 (3), 1439–1443. 10.1063/1.1637705.

[ref24] Moniruzzaman M.; Winey K. I. Polymer Nanocomposites Containing Carbon Nanotubes. Macromolecules 2006, 39 (16), 5194–5205. 10.1021/ma060733p.

[ref25] Banerjee J.; Dutta K. Melt-Mixed Carbon Nanotubes/Polymer Nanocomposites. Polym. Compos. 2019, 40 (12), 4473–4488. 10.1002/pc.25334.

[ref26] Alig I.; Pötschke P.; Lellinger D.; Skipa T.; Pegel S.; Kasaliwal G. R.; Villmow T. Establishment Morphology and Properties of Carbon Nanotube Networks in Polymer Melts. Polymer 2012, 53 (1), 4–28. 10.1016/J.POLYMER.2011.10.063.

[ref27] Longley L.; Collins S. M.; Zhou C.; Smales G. J.; Norman S. E.; Brownbill N. J.; Ashling C. W.; Chater P. A.; Tovey R.; Schönlieb C. B.; Headen T. F.; Terrill N. J.; Yue Y.; Smith A. J.; Blanc F.; Keen D. A.; Midgley P. A.; Bennett T. D. Liquid Phase Blending of Metal-Organic Frameworks. Nat. Commun. 2018, 9 (1), 2135 10.1038/s41467-018-04553-6. 29907760 PMC6004012

[ref28] Watcharatpong T.; Crespy D.; Kadota K.; Wang S.-M.; Kongpatpanich K.; Horike S. Alloying One-Dimensional Coordination Polymers To Create Ductile Materials. J. Am. Chem. Soc. 2024, 146, 23412–23416. 10.1021/jacs.4c06537. 39134058 PMC11345753

[ref29] Panda T.; Horike S.; Hagi K.; Ogiwara N.; Kadota K.; Itakura T.; Tsujimoto M.; Kitagawa S. Mechanical Alloying of Metal–Organic Frameworks. Angew. Chem., Int. Ed. 2017, 56 (9), 2413–2417. 10.1002/anie.201612587. 28112472

[ref30] Krishna A.; Gottis S.; Nazeeruddin M. K.; Sauvage F. Mixed Dimensional 2D/3D Hybrid Perovskite Absorbers: The Future of Perovskite Solar Cells?. Adv. Funct. Mater. 2019, 29 (8), 1806482 10.1002/adfm.201806482.

[ref31] Grancini G.; Nazeeruddin M. K. Dimensional Tailoring of Hybrid Perovskites for Photovoltaics. Nat. Rev. Mater. 2018, 4 (1), 4–22. 10.1038/s41578-018-0065-0.

[ref32] Wu G.; Liang R.; Zhang Z.; Ge M.; Xing G.; Sun G.; Wu G.; Liang R.; Zhang Z.; Ge M.; Xing G.; Sun G. 2D Hybrid Halide Perovskites: Structure, Properties, and Applications in Solar Cells. Small 2021, 17 (43), 2103514 10.1002/SMLL.202103514. 34590421

[ref33] Dou L.; Wong A. B.; Yu Y.; Lai M.; Kornienko N.; Eaton S. W.; Fu A.; Bischak C. G.; Ma J.; Ding T.; Ginsberg N. S.; Wang L.-W.; Alivisatos A. P.; Yang P. Atomically Thin Two-Dimensional Organic-Inorganic Hybrid Perovskites. Science 2015, 349 (6255), 1518–1521. 10.1126/science.aac7660. 26404831

[ref34] Zhang F.; Lu H.; Tong J.; Berry J. J.; Beard M. C.; Zhu K. Advances in Two-Dimensional Organic–Inorganic Hybrid Perovskites. Energy Environ. Sci. 2020, 13 (4), 1154–1186. 10.1039/C9EE03757H.

[ref35] Shaw B. K.; Hughes A. R.; Ducamp M.; Moss S.; Debnath A.; Sapnik A. F.; Thorne M. F.; McHugh L. N.; Pugliese A.; Keeble D. S.; Chater P.; Bermudez-Garcia J. M.; Moya X.; Saha S. K.; Keen D. A.; Coudert F.-X.; Blanc F.; Bennett T. D. Melting of Hybrid Organic–Inorganic Perovskites. Nat. Chem. 2021, 13 (8), 778–785. 10.1038/s41557-021-00681-7. 33972755

[ref36] Shaw B. K.; Castillo-Blas C.; Thorne M. F.; Ríos Gómez M. L.; Forrest T.; Lopez M. D.; Chater P. A.; McHugh L. N.; Keen D. A.; Bennett T. D. Principles of Melting in Hybrid Organic–Inorganic Perovskite and Polymorphic ABX _3_ Structures. Chem. Sci. 2022, 13 (7), 2033–2042. 10.1039/D1SC07080K. 35308849 PMC8849004

[ref37] Li T.; Dunlap-Shohl W. A.; Reinheimer E. W.; Le Magueres P.; Mitzi D. B. Melting Temperature Suppression of Layered Hybrid Lead Halide Perovskites *via* Organic Ammonium Cation Branching. Chem. Sci. 2019, 10 (4), 1168–1175. 10.1039/C8SC03863E. 30774915 PMC6349064

[ref38] Li T.; Dunlap-Shohl W. A.; Han Q.; Mitzi D. B. Melt Processing of Hybrid Organic–Inorganic Lead Iodide Layered Perovskites. Chem. Mater. 2017, 29 (15), 6200–6204. 10.1021/acs.chemmater.7b02363.

[ref39] Mitzi D. B.; Medeiros D. R.; DeHaven P. W. Low-Temperature Melt Processing of Organic–Inorganic Hybrid Films. Chem. Mater. 2002, 14 (7), 2839–2841. 10.1021/cm020264f.

[ref40] Wang W.; Liu C. D.; Fan C. C.; Fu X. B.; Jing C. Q.; Jin M. L.; You Y. M.; Zhang W. Rational Design of 2D Metal Halide Perovskites with Low Congruent Melting Temperature and Large Melt-Processable Window. J. Am. Chem. Soc. 2024, 146 (13), 9272–9284. 10.1021/jacs.4c00768. 38517743

[ref41] Ye C.; McHugh L. N.; Chen C.; Dutton S. E.; Bennett T. D. Glass Formation in Hybrid Organic-Inorganic Perovskites. Angew. Chem., Int. Ed. 2023, 62 (28), e202302406 10.1002/anie.202302406. 37012204

[ref42] Mitzi D. B.; Dimitrakopoulos C. D.; Rosner J.; Medeiros D. R.; Xu Z.; Noyan C. Hybrid Field-Effect Transistor Based on a Low-Temperature Melt-Processed Channel Layer. Adv. Mater. 2002, 14 (23), 1772–1776. 10.1002/1521-4095(20021203)14:23<1772::AID-ADMA1772>3.0.CO;2-Y.

[ref43] Singh A.; Jana M. K.; Mitzi D. B. Reversible Crystal–Glass Transition in a Metal Halide Perovskite. Adv. Mater. 2021, 33 (3), 2005868 10.1002/adma.202005868. 33283383

[ref44] Bennett T. D.; Yue Y.; Li P.; Qiao A.; Tao H.; Greaves N. G.; Richards T.; Lampronti G. I.; Redfern S. A. T.; Blanc F.; Farha O. K.; Hupp J. T.; Cheetham A. K.; Keen D. A. Melt-Quenched Glasses of Metal–Organic Frameworks. J. Am. Chem. Soc. 2016, 138 (10), 3484–3492. 10.1021/jacs.5b13220. 26885940

[ref45] Bennett T. D.; Horike S. Liquid, glass and amorphous solid states of coordination polymers and metal–organic frameworks. Nat. Rev. Mater. 2018, 3 (11), 431–440. 10.1038/s41578-018-0054-3.

[ref46] Ma N.; Horike S. Metal-Organic Network-Forming Glasses. Chem. Rev. 2022, 122, 4163–4203. 10.1021/acs.chemrev.1c00826. 35044749

[ref47] Singh A.; Kim Y.; Henry R.; Ade H.; Mitzi D. B. Study of Glass Formation and Crystallization Kinetics in a 2D Metal Halide Perovskite Using Ultrafast Calorimetry. J. Am. Chem. Soc. 2023, 145 (33), 18623–18633. 10.1021/jacs.3c06342. 37552801

[ref48] Pokroy B.; Fitch A. N.; Zolotoyabko E. The Microstructure of Biogenic Calcite: A View by High-Resolution Synchrotron Powder Diffraction. Adv. Mater. 2006, 18 (18), 2363–2368. 10.1002/adma.200600714.

[ref49] Zolotoyabko E. Basic Concepts of X-Ray Diffraction; Wiley VCH: Weinheim, 2014.

[ref50] Bonadio A.; Escanhoela C. A.; Sabino F. P.; Sombrio G.; de Paula V. G.; Ferreira F. F.; Janotti A.; Dalpian G. M.; Souza J. A. Entropy-Driven Stabilization of the Cubic Phase of MaPbI _3_ at Room Temperature. J. Mater. Chem. A 2021, 9 (2), 1089–1099. 10.1039/D0TA10492B.

[ref51] Pool V. L.; Dou B.; Van Campen D. G.; Klein-Stockert T. R.; Barnes F. S.; Shaheen S. E.; Ahmad M. I.; van Hest M. F. A. M.; Toney M. F. Thermal Engineering of FAPbI3 Perovskite Material via Radiative Thermal Annealing and in Situ XRD. Nat. Commun. 2017, 8 (1), 14075 10.1038/ncomms14075. 28094249 PMC5247577

[ref52] Jana M. K.; Song R.; Liu H.; Khanal D. R.; Janke S. M.; Zhao R.; Liu C.; Valy Vardeny Z.; Blum V.; Mitzi D. B. Organic-to-Inorganic Structural Chirality Transfer in a 2D Hybrid Perovskite and Impact on Rashba-Dresselhaus Spin-Orbit Coupling. Nat. Commun. 2020, 11 (1), 4699 10.1038/s41467-020-18485-7. 32943625 PMC7499302

[ref53] Castañeda R.; Lindeman S. V.; Krivoshein A. V.; Metta-Magaña A. J.; Chen Y.; Timofeeva T. V. Remarkable Similarity of Molecular Packing in Crystals of Racemic and Enantiopure 2-Phenylpropionamide: *Z*′ = 4 Structures, Molecular Disorder, and the Formation of a Partial Solid Solution. Cryst. Growth Des. 2022, 22 (7), 4592–4600. 10.1021/acs.cgd.2c00509. PMC11068035 38707789

[ref54] Zhao Y.; Zhao J.; Guo Y.; Zhao J.; Feng J.; Geng Y.; Yang J.; Gao H.; Yuan M.; Jiang L.; Wu Y. Reversible Phase Transition for Switchable Second Harmonic Generation in 2D Perovskite Microwires. SmartMat 2022, 3 (4), 657–667. 10.1002/smm2.1120.

[ref55] Shang J.; Liu S.; Ma X.; Lu L.; Deng Y. A New Route of CO2 Catalytic Activation: Syntheses of N-Substituted Carbamates from Dialkyl Carbonates and Polyureas. Green Chem. 2012, 14 (10), 2899 10.1039/c2gc36043h.

[ref56] Singh A.; Dayton D.; Ladd D. M.; Reuveni G.; Paluch P.; Montagne L.; Mars J.; Yaffe O.; Toney M.; Manjunatha Reddy G. N.; Mitzi D. B. Local Structure in Crystalline, Glass and Melt States of a Hybrid Metal Halide Perovskite. J. Am. Chem. Soc. 2024, 146, 25656–25668. 10.1021/jacs.4c07411. 39230963

[ref57] Makuła P.; Pacia M.; Macyk W. How To Correctly Determine the Band Gap Energy of Modified Semiconductor Photocatalysts Based on UV-Vis Spectra. J. Phys. Chem. Lett. 2018, 9 (23), 6814–6817. 10.1021/acs.jpclett.8b02892. 30990726

[ref58] Lang A.; Polishchuk I.; Seknazi E.; Feldmann J.; Katsman A.; Pokroy B. Bioinspired Molecular Bridging in a Hybrid Perovskite Leads to Enhanced Stability and Tunable Properties. Adv. Funct. Mater. 2020, 30 (42), 2005136 10.1002/adfm.202005136.

[ref59] Elmelund T.; Scheidt R. A.; Seger B.; Kamat P. V. Bidirectional Halide Ion Exchange in Paired Lead Halide Perovskite Films with Thermal Activation. ACS Energy Lett. 2019, 4 (8), 1961–1969. 10.1021/acsenergylett.9b01280.

[ref60] Jiang H.; Cui S.; Chen Y.; Zhong H. Ion Exchange for Halide Perovskite: From Nanocrystal to Bulk Materials. Nano Sel. 2021, 2 (11), 2040–2060. 10.1002/nano.202100084.

[ref61] Baranovskii S. D. Theoretical Description of Charge Transport in Disordered Organic Semiconductors. Phys. Status Solidi B 2014, 251 (3), 487–525. 10.1002/pssb.201350339. 25671376

[ref62] Stutzmann M.; Jackson W. B.; Tsai C. C. Light-Induced Metastable Defects in Hydrogenated Amorphous Silicon: A Systematic Study. Phys. Rev. B: Condens. Matter Mater. Phys. 1985, 32 (1), 23–47. 10.1103/PhysRevB.32.23. 9936636

[ref63] Wu Y.; Huang H. W.; Olah G. A. Method of Oriented Circular Dichroism. Biophys. J. 1990, 57 (4), 797–806. 10.1016/S0006-3495(90)82599-6. 2344464 PMC1280780

[ref64] Wormell P.; Rodger A. Absorbance Spectroscopy: Spectral Artifacts and Other Sources of Error. In Encyclopedia of Biophysics; Springer Berlin Heidelberg: Berlin, Heidelberg, 2018; pp 1–4.

[ref65] Miles A. J.; Wallace B. A. Circular Dichroism Spectroscopy of Membrane Proteins. Chem. Soc. Rev. 2016, 45 (18), 4859–4872. 10.1039/C5CS00084J. 27347568

