# Supplementary material for: Melt Alloying of Two-Dimensional Hybrid Perovskites: Composition-Dependence of Thermal and Optical Properties
Source: J Am Chem Soc. 2024 Nov 22;146(49):33945–55. doi: 10.1021/jacs.4c12697 (PMC11638950; doi:10.1021/jacs.4c12697)
Supplement: Supplementary file 1 — ja4c12697_si_001.pdf [file ja4c12697_si_001.pdf]

Supporting Information

Melt Alloying of Two-Dimensional Hybrid Perovskites: Composition-Dependence of Thermal and Optical Properties

Arad Lang[*,1]*, Celia Chen*[1]*, Chumei Ye*[1]*, Lauren N. McHugh*[2]*, Xian Wei Chua*[3,4]*, Samuel D. Stranks*[3,4]*, Siân E. Dutton*[3]*, Thomas D. Bennett*[1]

[1] Department of Materials Science and Metallurgy, University of Cambridge, 27 Charles Babbage Road, Cambridge CB3 0FS, United Kingdom.

[2] Department of Chemistry, University of Liverpool, Crown Street, Liverpool, L69 7ZD, United Kingdom.

[3] Cavendish Laboratory, University of Cambridge, JJ Thomson Avenue, Cambridge, CB3 0HE, United Kingdom.

[4] Department of Chemical Engineering and Biotechnology, University of Cambridge, Philippa Fawcett Drive, Cambridge, CB3 0AS, United Kingdom.

* al2213@cam.ac.uk

Table S1. 2D-HOIPs amounts and temperatures.

$X_{1\text{-MHA}}$ [% at]	$(1\text{-MHA})_2PbI_4$ [mg]	$(S\text{-NEA})_2PbBr_4$ [mg]	$T_{m,exp}$† [°C]	$T_{x,exp}$† [°C]
**0**	0.0	250.0	185	160
**10**	23.7	196.1	185	132
**30**	71.2	152.8	185	103
**50**	118.8	109.0	185	80
**70**	165.8	65.4	185	84
**90**	213.4	21.9	185	100
**100**	250.0	0.0	185	157

† $T_{m,exp}$ and $T_{x,exp}$ are the temperatures used for melting and recrystallisation of the samples, respectively (during the XRD measurement). They are *higher* than the ones calculated based on the DSC measurements. $T_{m,exp}$ and $T_{x,exp}$ were selected based on the DSC profiles of the samples (Figure 2a,d in the main text), to allow melting *or* recrystallisation (without remelting), respectively.

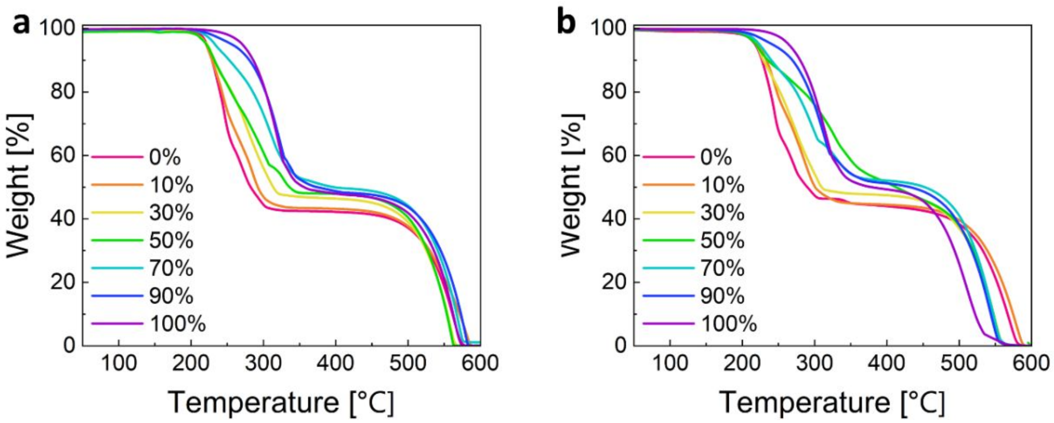

Figure S1. TGA curves of the blends: (a) as-is, and (b) after melting.

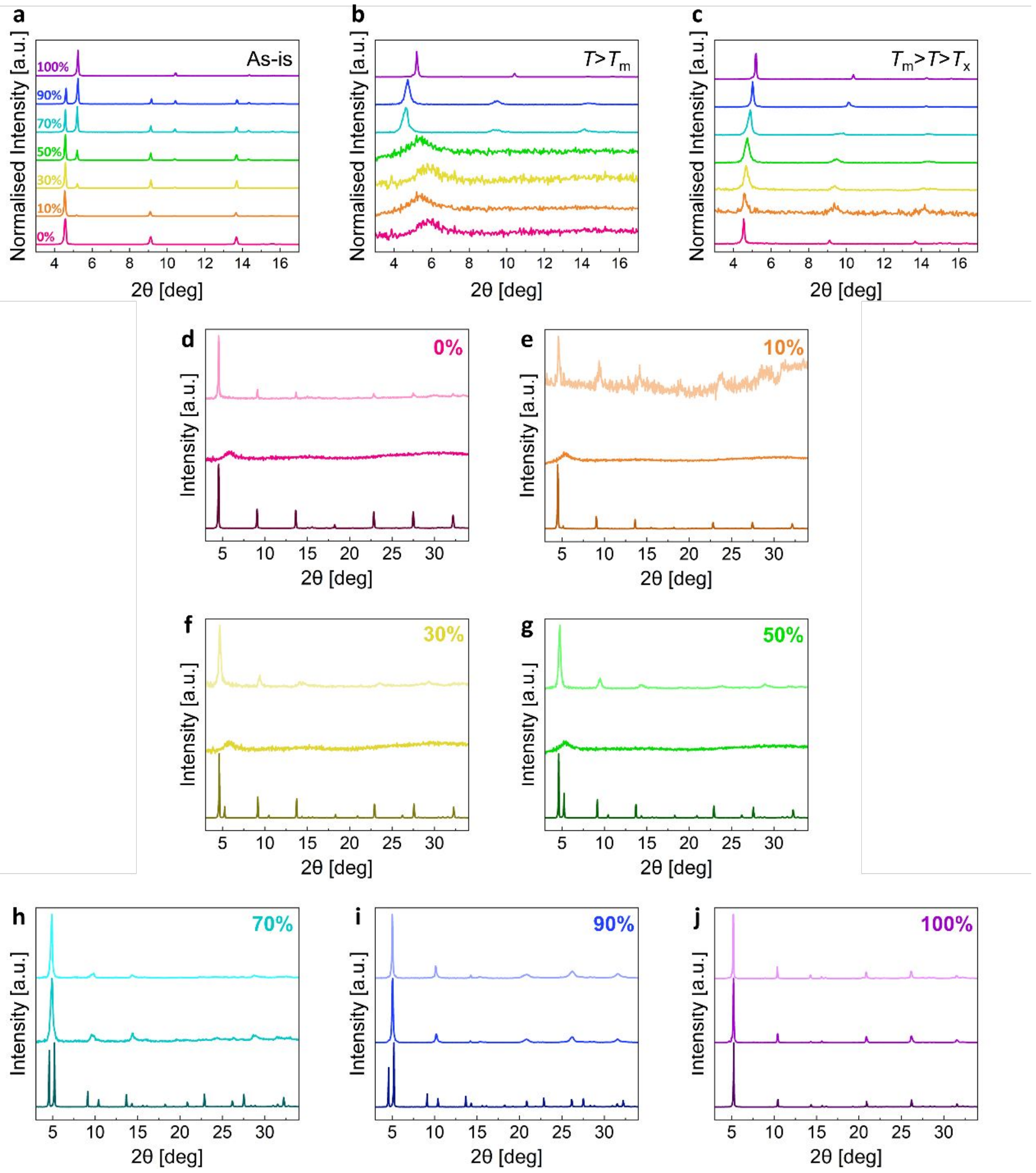

Figure S2. Full XRD patterns of the blends: (a) as-is, (b) after melting, (c) after recrystallisation, (d) $X_{1\text{-MHA}}$=0% mol, (e) $X_{1\text{-MHA}}$=10% mol, (f) $X_{1\text{-MHA}}$=30% mol, (g) $X_{1\text{-MHA}}$=50% mol, (h) $X_{1\text{-MHA}}$=70% mol, (i) $X_{1\text{-MHA}}$=90% mol, (j) $X_{1\text{-MHA}}$=100% mol.

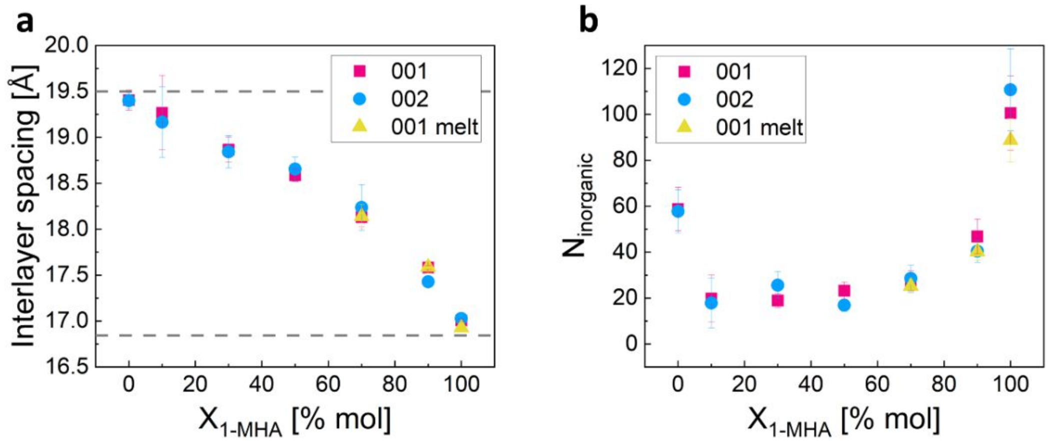

Figure S3. (a) Inorganic interlayer spacing, and (b) average number of inorganic layers in a crystal, calculated using Equations (1) and (2), based on the diffraction patterns of the blends. The magenta squares represent data obtained based on the (001) reflection (2θ=4-5º), while the cyan circles represent data obtained based on the (002) reflections (2θ=9-10º). For comparison, the yellow triangles represent the data calculated based on the (001) reflection of the crystalline samples after melting.

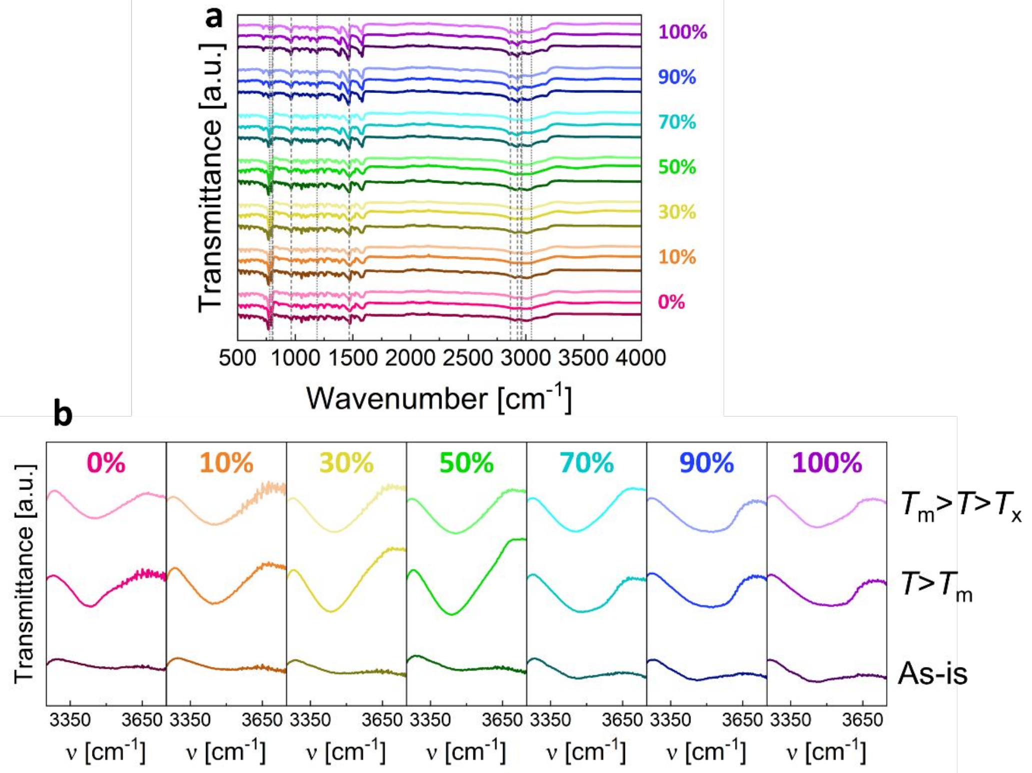

Figure S4. (a) FTIR spectra of the blends. In each set of spectra, corresponding to a different blend ratio (as indicated on the right), the stacking is as follows: bottom – as-is blend; middle – melt after melting; top – blend after recrystallisation. The main absorbance peaks of the organic molecules were assigned based on the Spectral Database for Organic Compounds (SDBS), and marked as follows: dashed lines – 1-MHA; dotted lines – S-NEA. (b) Zoom-in on the IR absorbance peak of each sample, corresponds to free amine groups.

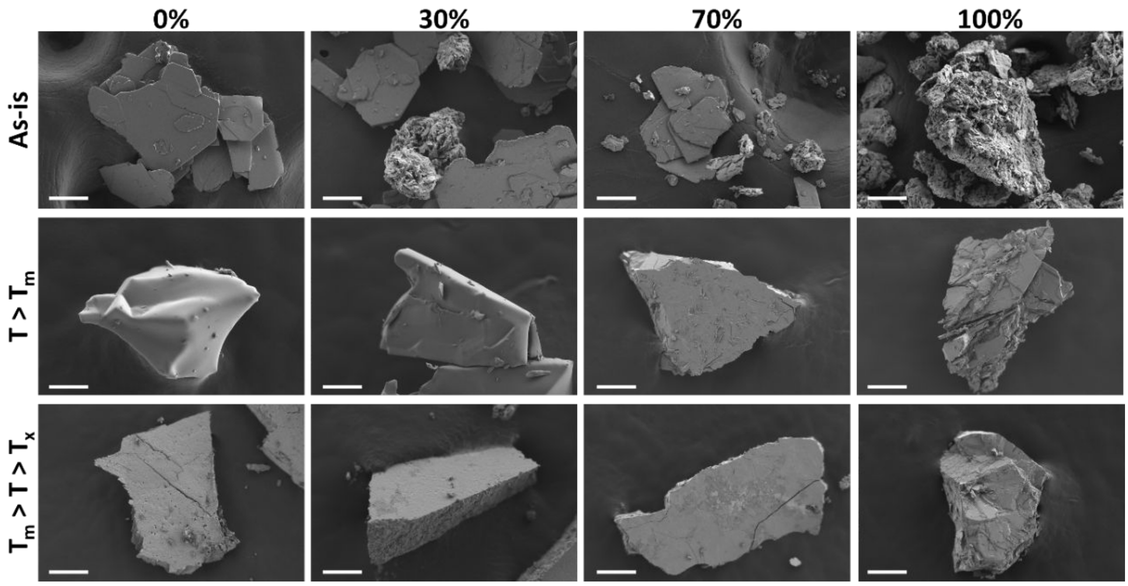

Figure S5. Secondary electrons (SE) micrographs of selected samples, at different stages: as-is (top row), after melting (middle row), and after recrystallisation (bottom row). The (S-NEA)$_2$PbBr$_4$ plates and (1-MHA)^{2}PbI^4 aggregates are clearly visible in the as-is samples. Scalebars: 50 µm.

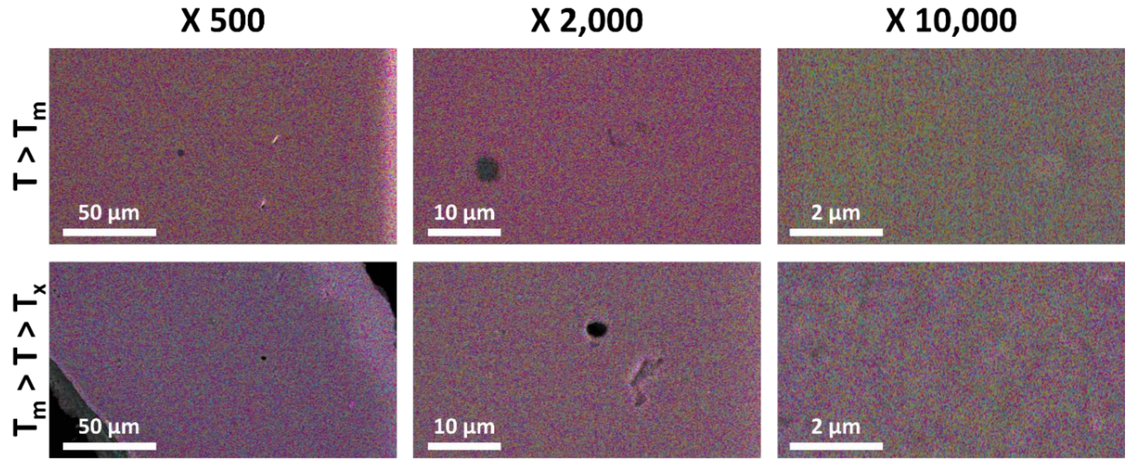

Figure S6. High-magnification EDS elemental maps, presented on top of the BSE micrographs, of $X_{1\text{-MHA}}$ = 50% mol. Blend, after melting (top row) and after recrystallisation (bottom row). The colours of each element are as follows: Pb – yellow; Br – magenta; I – cyan.

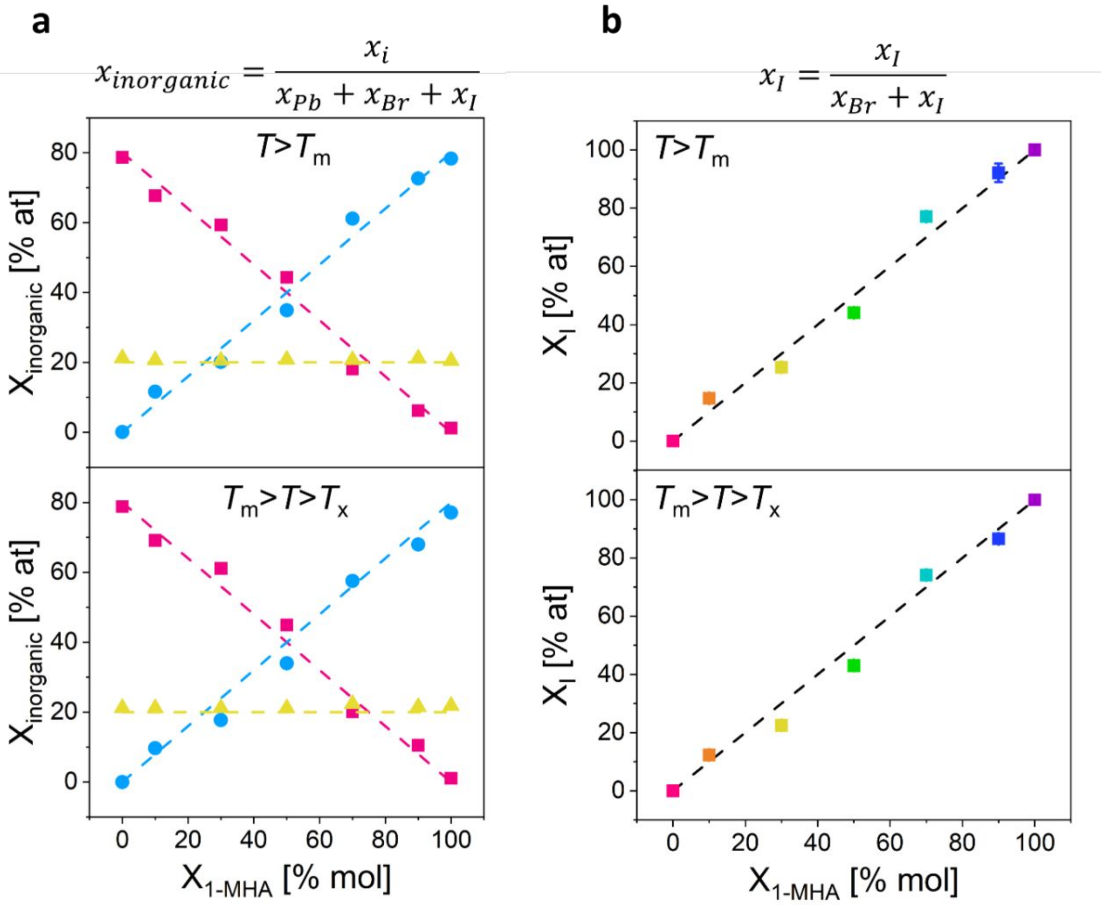

Figure S7. EDS quantitative analysis of the blends after melting: (a) the concentration of Pb (yellow), Br (magenta), and I (cyan) relative to the total inorganic content in the sample. (b) The concentration of I relative to the total halide content in the sample. The top plot of each pair represents the sample after melting, while the bottom plot represents the samples after recrystallisation. The dashed lines represent the theoretical expected trend, determined by the stoichiometry of the crystals and $X_{1\text{-MHA}}$ of the blends.

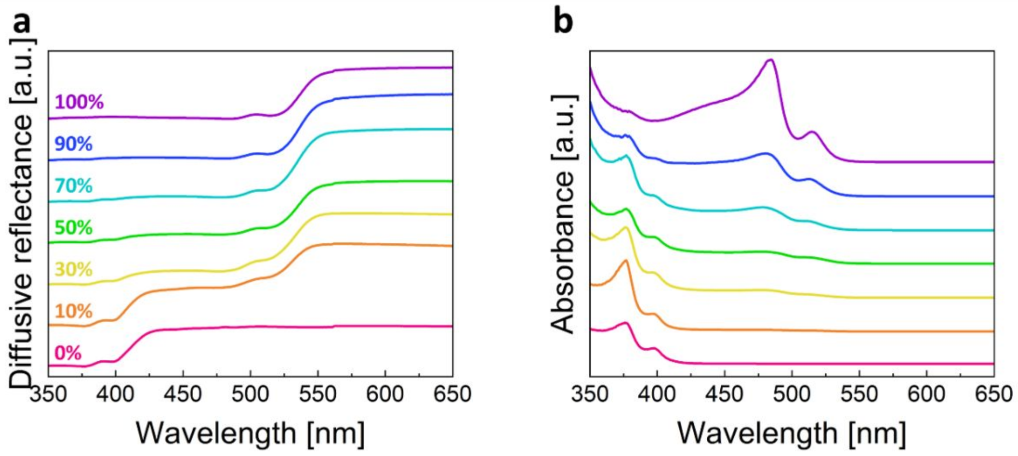

Figure S8. (a) Diffuse reflectance, and (b) absorbance UV-Vis spectra of the as-is blends.

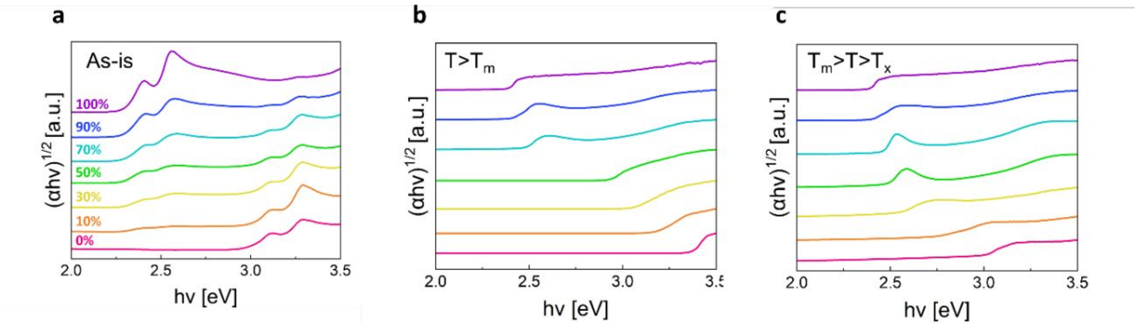

Figure S9. Tauc plots of the blends (a) as-is, (b) after melting, and (c) after recrystallisation.

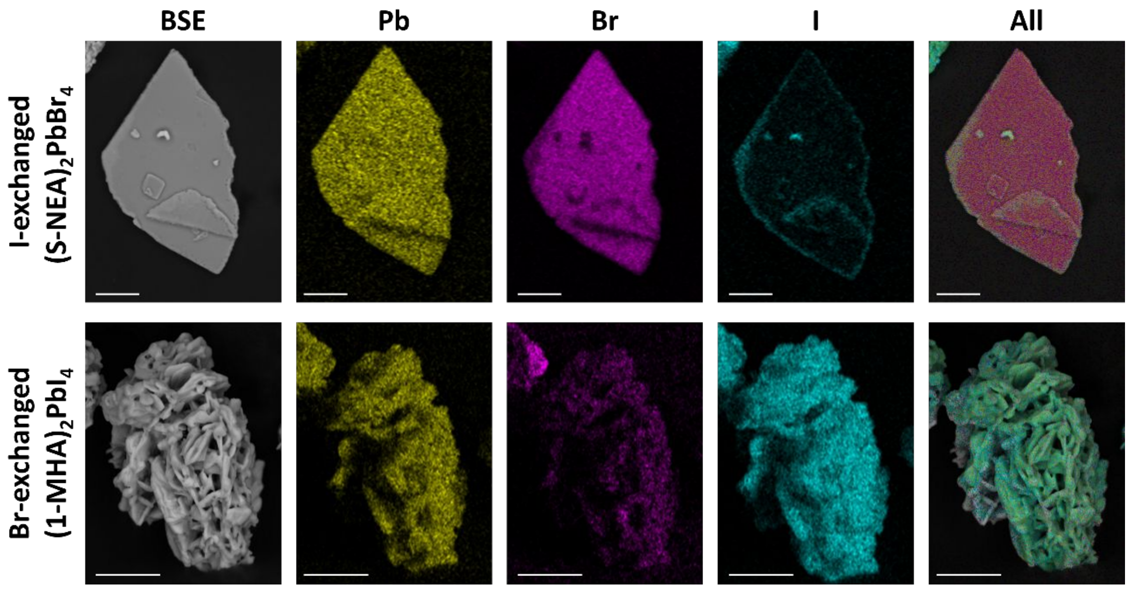

Figure S10. EDS elemental maps of two selected (S-NEA)$_2$PbBr$_4$ (top row) and (1-MHA)$_2$PbI$_4$ (bottom row) crystals from the sample with $X_{1\text{-MHA}}$=50% mol. Each row presents, from left to right: back-scattered electrons micrograph, Pb map, Br map, I map, and all of the above layered together. The clear I signal in the (S-NEA)$_2$PbBr$_4$ crystal, as well as the Br signal in the (1-MHA)$_2$PbI$_4$ crystal, confirms the occurrence of halide exchange.

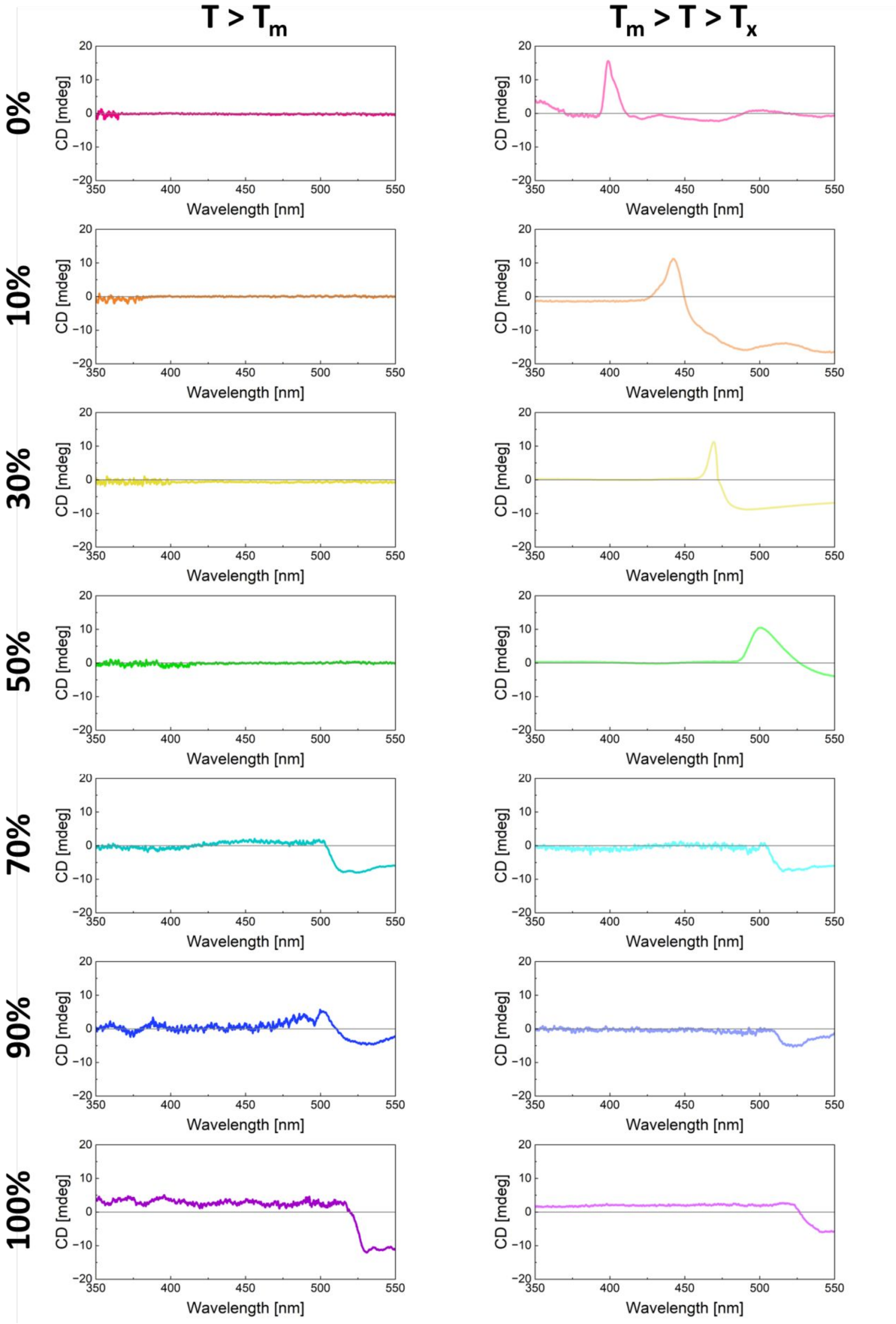

Figure S11. CD spectra of the melted (left) and recrystallized (right) samples. The horizontal grey lines are inserted for reference, representing a zero CD response.
